# Pimarane-Type Diterpenes with Anti-Inflammatory Activity from Arctic-Derived Fungus *Eutypella* sp. D-1

**DOI:** 10.3390/md21100541

**Published:** 2023-10-18

**Authors:** Yaodong Ning, Shi Zhang, Te Zheng, Yao Xu, Song Li, Jianpeng Zhang, Binghua Jiao, Yun Zhang, Zengling Ma, Xiaoling Lu

**Affiliations:** 1 Department of Biochemistry and Molecular Biology, College of Basic Medical Sciences, Naval Medical University, Shanghai 200433, China; 2 College of Life and Environmental Sciences, Wenzhou University, Wenzhou 325000, China; 3 Biology Institute, Qilu University of Technology, Shandong Academy of Sciences, Jinan 250000, China

**Keywords:** *Eutypella* sp. D-1, diterpene, anti-inflammatory, zebrafish model

## Abstract

The Arctic-derived fungus *Eutypella* sp. D-1 can produce numerous secondary metabolites, and some compounds exhibit excellent biological activity. Seven pimarane-type diterpenes, including three new compounds eutypellenone F (**1**), libertellenone Y (**2**), and libertellenone Z (**3**), and four known compounds (**4**–**7**), were isolated from fermentation broth of *Eutypella* sp. D-1 by the OSMAC strategy of adding ethanol as a promoter in the culture medium. Compound **2** has a rare tetrahydrofuran-fused pimarane diterpene skeleton. The anti-inflammatory activity of all compounds was evaluated. Compounds **3**–**6** showed a significant inhibitory effect on cell NO release at 10 μmol/L by in vitro experiments, of which **3**–**5** had inhibitory rates over 60% on nitric oxide (NO) release. Subsequently, the anti-inflammatory activity of **3**–**5** was evaluated based on a zebrafish model, and the results showed that **3** had a significant inhibitory effect on inflammatory cells migration at 40 μmol/L, while **4** and **5** had a significant inhibitory effect at 20 μmol/L. Moreover, compounds **3**–**5** have the same conjugated double bond structure, which may be an important group for these compounds to exert anti-inflammatory activity.

## 1. Introduction

Inflammation is a reaction caused by organism damage, which is a way for higher animals to combat infection and trauma. The inflammation can remove harmful factors and damaged tissues and restore the organism to normal [1,2,3]. However, chronic and intense inflammation can participate in the pathological processes of many diseases, such as arthritis, tumors, and metabolic syndrome [4]. Therefore, the development of anti-inflammatory drugs possesses great significance. Currently, the commonly used anti-inflammatory drugs in the clinic are still insufficient, and both of them have obvious adverse reactions [5]. Consequently, there is an urgent need to search for some anti-inflammatory leading drugs with efficient and low toxicity.

Microorganisms can produce a wide variety of secondary metabolites (e.g., polyketides, terpenes, and non-ribosomal peptides), some of which show excellent biological activity, such as antibacterial, anti-tumor, and immunoregulation activities [6,7,8]. Due to the extreme living environment (e.g., bitter cold and hypoxia), polar fungi could induce the production of secondary metabolites with novel skeletons and rich biological activity [9,10]. *Eutypella* sp. D-1, a fungus isolated from high-latitude areas in the Arctic, can produce structurally diverse secondary metabolites; for instance, terpenes, cytochalasins, steroids, lactones, etc. [11,12,13].

**Figure 1:**
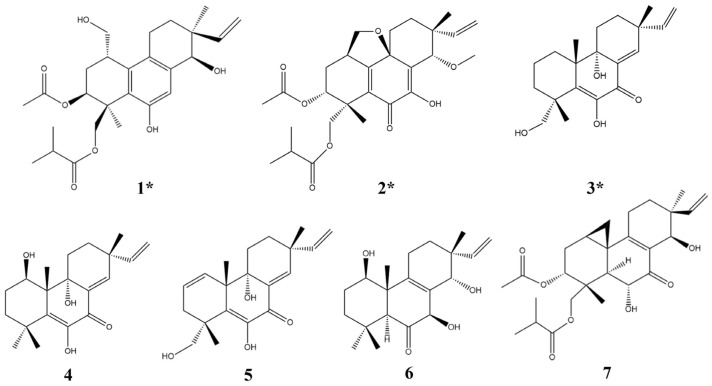
Structures of compounds **1**–**7** (* new compounds).

The pimarane-type diterpenes are the main secondary metabolites of *Eutypella* sp. D-1, some of which showed significant anti-tumor activity, such as libertellenone H [14,15,16]. Through further research, it was demonstrated that the addition of ethanol can prominently increase the yield of libertellenone H. Transcriptome analysis showed that the addition of ethanol could increase the expression of genes related to terpene biosynthesis and enhance the activity of rate-limiting enzymes related to the mevalonate pathway, which may promote an increment in the types and yield of terpenes in *Eutypella* sp. D-1 [17]. Consequently, in this study, seven pimarane-type diterpenes were obtained from *Eutypella* sp. D-1 fermentation broth by adding ethanol (Figure 1), including the three new compounds eutypellenone F (**1**), libertellenone Y (**2**), and libertellenone Z (**3**), with four known compounds (**4**–**7**). Meanwhile, based on the extensive biological activities of terpene, the anti-inflammatory activity is also evaluated in vivo and in vitro.

## 2. Results and Discussion

### 2.1. Structural Analysis of Compounds

Eutypellenone F (**1**) was isolated as a white powder. The molecular formula was determined to be C_26_H_36_O_7_ by high-resolution electrospray ionization mass spectroscopy (HRESIMS), implying nine degrees of unsaturation. The ultraviolet (UV) spectrum exhibited absorption at *λ*_max_ 206 nm. The ^1^H-NMR and ^13^C-NMR data are shown in Table 1 and Supplementary Materials Figures S1 and S2. The ^1^H-NMR data display three characteristic methyl proton signals at *δ*_H_ 1.18 (3H, s), 1.54 (3H, s), and 2.17 (3H, s); one olefinic proton at *δ*_H_ 6.86 (1H, s) and a terminal vinyl group at *δ*_H_ 5.83 (1H, q, *J* = 7.24 Hz) and 5.21 (2H, m). Analysis of the ^13^C-NMR data and DEPT spectrum revealed a total of 26 carbons including five methyls, six methylenes, six hypomethylenes, and nine quaternary carbon signals. Four carbons connecting oxygen atom at *δ*_C_ 63.96, 65.42, 74.90, and 75.42, and two ketone carbonyl signals at *δ*_C_ 171.50 and 176.80 could be observed from the ^13^C-NMR.

**Figure 2:**
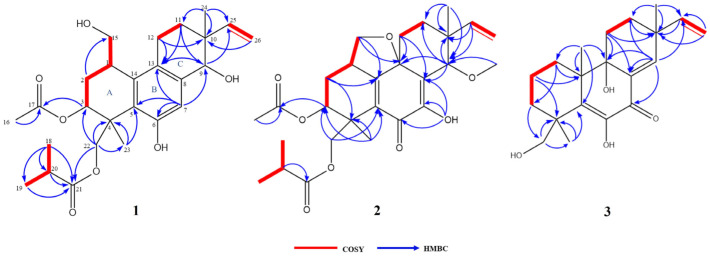
COSY and key HMBC correlations of compounds **1**–**3**.

The COSY correlations of H-1/H-2b, H-1/H-15, H-2b/H-3, H-11a/H-12, H-18/H-20, and H-19/H-20 revealed the presence of four isolated spin systems: C-15/C-1/C-2/C-3, C-11/C-12, C-25/C-26, and C-18/C-20/C-19 (Figure 2). The diagnostic HMBC correlations from H-9 to C-13/C-25, H-11a to C-9/C-12/C-13, and H-12 to C-10/C-11/C-13 indicated the existence of a ring C. In addition, the correlations from H-18 to C-19/C-20/C-21, H-23 to C-3/C-4/C-5/C-22, and H-24 to C-9/C-10/C-11/C-25 indicated the presence of a pimarane-type diterpene. Due to the presence of four oxygenated carbon signals, the oxygenated carbon at C-9/C-15 had to be substituted with a hydroxy group to satisfy the molecular formula (Figure 2). All of these spectroscopic characteristics revealed that **1** is a member of the libertellenone class [14].

**Figure 3:**
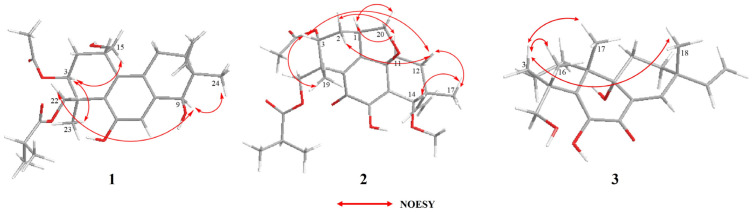
Key NOESY correlations of compounds **1**–**3**.

**Figure 4:**
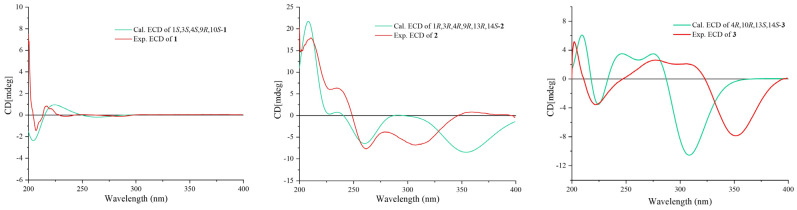
Calculated and experimental ECD spectra of compounds **1**–**3**.

Once the planar structure was established, its relative configuration was addressed by NOESY experiments (Figure 3). The NOESY cross-peaks of H-3/H-15 and H-3/H-23 indicated the same orientation of these protons. Correspondingly, H-9/H-22a/H-24 oriented in the other direction could be obtained. Therefore, the relative structure was determined. In order to determine the whole absolute configurations of **1**, the quantum chemical electronic circular dichroism (ECD) calculation method was employed. The negative Cotton effect (CE) at 205 nm in the calculated spectrum of the 1*S*, 3*S*, 4*S*, 9*R*, 10*S* enantiomer approximately matched the CE observed in the experimental ECD spectrum of **1** (Figure 4), enabling assignment of the absolute configurations of **1** as shown.

Libertellenone Y (**2**) was isolated as a yellow oil. The molecular formula was determined to be C_27_H_36_O_8_ by HRESIMS, implying 10 degrees of unsaturation. The UV spectrum exhibited absorption at *λ*_max_ 205 and 250 nm. The ^1^H-NMR and ^13^C-NMR data are shown in Table 1 and Supplementary Materials Figures S11 and S12. The ^1^H-NMR data display three characteristic methyl proton signals at *δ*_H_ 1.20 (3H, s), 1.36 (3H, s), and 2.00 (3H, s). The detailed analysis of the ^13^C-NMR data and DEPT spectrum revealed a total of 27 carbons, including five methyls, six methylenes, five hypomethylenes, and ten quaternary carbons.

The COSY and HMBC correlations revealed that compound **2** is similar to eutypellenoid B [15], except for the replacement of a hydroxy with a methoxy group at the C-14 position (Figure 2). Once the planar structure was established, its relative configuration was addressed by NOESY experiments (Figure 3). The NOESY cross-peaks of H-1/H-11a, H-1/H-20a, and H-11a/H-2a indicated the same orientation of these protons. Correspondingly, H-2b/H-3/H-12b/H-14/H-17/H-19 oriented in the other direction could be obtained. Therefore, the relative structure was determined. In order to determine the whole absolute configurations of **2**, the ECD calculation method was employed. Likewise, the experimental ECD spectrum of **2** was in good agreement with the calculated spectrum for 1*R*, 3*R*, 4*R*, 9*R*, 13*R*, 14*S* (Figure 4).

Libertellenone Z (**3**) was isolated as a yellow oil. The molecular formula was determined to be C_20_H_28_O_4_ by HRESIMS, implying seven degrees of unsaturation. The UV spectrum of **3** exhibited absorption at *λ*_max_ 266 nm and 324 nm. The ^1^H-NMR and ^13^C-NMR data of **3** are shown in Table 1 and Supplementary Materials Figures S19 and S20. The ^1^H-NMR data of **3** display three characteristic methyl proton signals at *δ*_H_ 1.12 (3H, s), 1.17 (3H, s), and 1.20 (3H, s); one olefinic proton at *δ*_H_ 7.04 (1H, d, *J* = 1.86 Hz), and a terminal vinyl group at *δ*_H_ 5.88 (1H, q, *J* = 6.41 Hz) and 5.08 (2H, m). Detailed analysis of the ^13^C-NMR and DEPT spectrum revealed a total of 20 carbons, including three methyls, seven methylenes, two hypomethylenes, and eight quaternary carbons. Two carbons connecting oxygen atoms at *δ*_C_ 70.49 and 74.86, and one ketone carbonyl signal at *δ*_C_ 181.80 could be observed from the ^13^C-NMR.

The COSY and HMBC correlations revealed that the structure of **3** is similar to libertellenone A [18], except for the replacement of a hydroxy with a hydrogen atom at the C-1 position, and the replacement of a hydrogen atom with a hydroxy at C-15 position (Figure 2). Once the planar structure of **3** was established, its relative configuration was addressed by NOESY experiments (Figure 3). The NOESY cross-peaks of H-3b/H-16, H-3b/H-17, and H-3b/H-18 indicated the same orientation of these protons. Therefore, the relative structure of **3** was determined. In order to determine the whole absolute configurations of **3**, the ECD calculation method was employed. The positive CE at 204 nm, and the negative CE at 220 nm and 352 nm in the calculated spectrum of the 4*R*, 10*R*, 13*S*, 14*S* enantiomer approximately matched the CE observed in the experimental ECD spectrum of **3** (Figure 4), which enabled the assignment of the absolute configurations of **3** as shown.

In addition, four known compounds (**4**–**7**) were also isolated as metabolites of *Eutypella* sp. D-1 (Figure 1). These compounds were identified by comparing their spectral data with spectroscopic data reported in the corresponding literature, and all of these belong to diterpenes of the pimarane type [13,14,18,19]. 

### 2.2. Evaluation of the Inhibitory Effect on the NO Release

**Figure 5:**
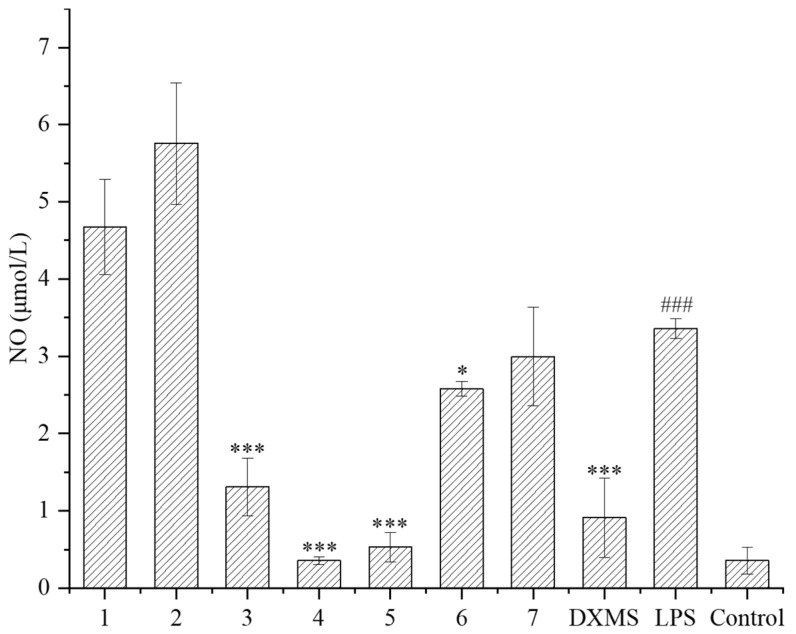
NO release of LPS-induced RAW264.7 inflammatory cells under compounds treatment (DXMS: dexamethasone group. The compounds group was compared with the LPS group, * *p* < 0.05, and *** *p* < 0.001. ^###^ *p* < 0.001 versus the control group).

The LPS-induced RAW264.7 cell was used as an inflammatory model. RAW264.7 macrophages can stimulate inflammatory reactions under the induction of LPS, and release various inflammatory mediators such as NO, TNF-α, and interleukin-6, which can be used to evaluate the anti-inflammatory activity of secondary metabolites. In this study, the inhibitory effects of compounds on the NO released from cells were evaluated. Firstly, the safe concentration of compounds was determined. Except for compound **6**, the safe concentration of all the other compounds was greater than 40 μmol/L, indicating that these compounds have little effect on cell proliferation at high concentration. Subsequently, the NO releasing was measured at 10 μmol/L of these compounds. It is shown that compounds **3**–**6** had a significant inhibitory effect on the NO releasing compared to the LPS treatment group (Figure 5), of which compounds **3**–**5** had a very noticeable inhibitory effect, close to or exceeding the effect of the positive drug dexamethasone (DXMS). Compounds **3**–**5** exhibited that strong inhibition rates of NO releasing exceeded 60%, and compound **4** had a maximum inhibition rate of 89%, which was already superior to the effect of the positive drug dexamethasone (72%) (Table 2).

### 2.3. Evaluation of Anti-Inflammatory Activity Based on Zebrafish Model

**Figure 6:**
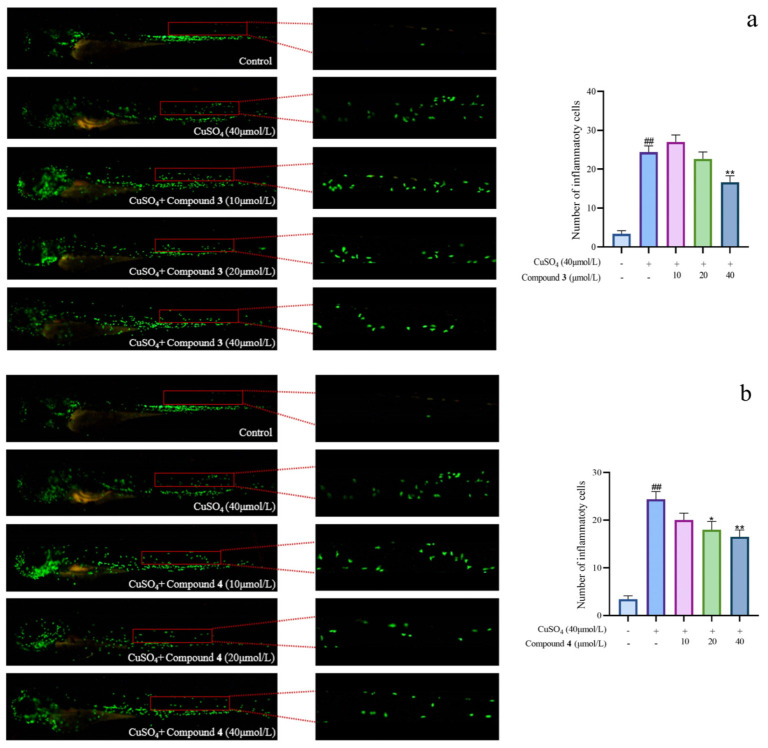

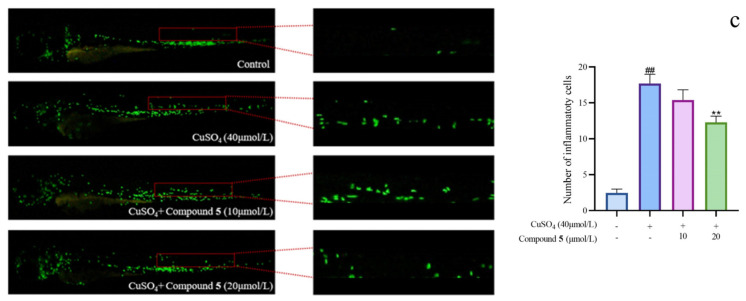
The migration of zebrafish inflammatory cells induced by CuSO_4_ ((**a**–**c**) represent the number of inflammatory cells that migrated to the lateral line in zebrafish treated with compound **3**–**5**, respectively. The compounds group was compared with the CuSO_4_ group, * *p* < 0.05, and ** *p* < 0.01. ^##^ *p* < 0.01 versus the control group).

**Figure 7:**
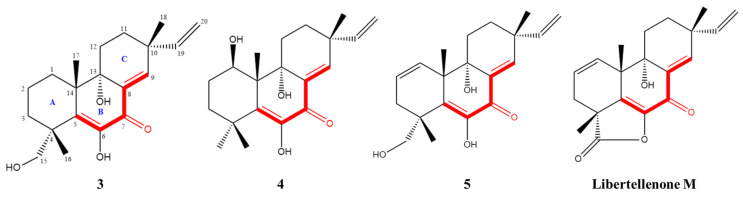
Compounds with conjugated double bond structure.

Given the significant anti-inflammatory activity of **3**–**5**, further in-depth research was conducted based on the zebrafish model. The effect of **3**–**5** on the migration of zebrafish inflammatory cells induced by CuSO_4_ was investigated. Compared with the control group, the number of inflammatory cells migrating to the lateral line of the zebrafish of the CuSO_4_ model group apparently increased, indicating the success of the acute inflammation model (Figure 6). Compared with the CuSO_4_ group, the compound **3** treatment group clearly reduced the number of inflammatory cells migrating to the lateral line of zebrafish at 40 μmol/L (Figure 6a), while compounds **4**–**5** at 20 μmol/L performed likewise (Figure 6b,c). According to the in vivo and in vitro activity experiment, it was observed that compounds **3**–**5** all have the same conjugated double bond structure at C-5/C-6 and C-8/C-9 positions (Figure 7), with the hydroxyl group at C-6 and carbonyl group at the C-7 position. It is speculated that the conjugated structure with the carbonyl and hydroxyl groups in the B and C ring is an important pharmacophore group for their anti-inflammatory activity. Meanwhile, a study reported that the compound Libertellenone M, with the same conjugated double bond structure, also has good anti-inflammatory activity, and researchers conducted in-depth research on its anti-inflammatory mechanism [20] (Figure 7). On the other hand, similar compounds (**1**–**2** and **6**–**7**) without this structure fragment do not have good anti-inflammatory activity (Figure 5).

## 3. Materials and Methods

### 3.1. General Experimrntal Procedures

Nuclear magnetic resonance (NMR) spectra were measured on a Bruker AMX-500 instrument (Bruker Biospin Corp., Billerica, MA, USA) at 500 MHz for ^1^H-NMR and 125 MHz for ^13^C-NMR. High-resolution electrospray ionization mass spectroscopy (HRESIMS) data were obtained on an Agilent 6210 LC/MSD TOF mass spectrometer (Agilent Technologies Inc. Lake Forest, CA, USA), and ultraviolet (UV) spectra were obtained on a UV-8000 spectrophotometer (Shanghai Metash instruments Co., Shanghai, China). Optical rotations were measured on an Anton Paar MCP 5500 (Anton Paar Co, Graz, Austria). Semi-preparative high-performance liquid chromatography (HPLC) was performed on a Waters 1525 separation module (Waters Corp., Milford, MA, USA) equipped with a Waters 2996 photodiode array detector (Waters Corp., Milford, MA, USA) by using YMC-Pack Pro C18 RS (5 µm) columns (YMC Co. Ltd., Kyoto, Japan). Purifications were conducted by Agilent LC1263 Infinity Ⅱ (Agilent Technologies Inc., CA, USA). Thin-layer chromatography analysis was run on HSGF254-precoated silica gel plates (10–40 mm, Yantai Chemical Plant, Yantai, China).

### 3.2. Fungal Strain

The fungus *Eutypella* sp. D-1 (CCTCC NO: M2013144) was isolated from the soil of London Island of Kongsfjorden of Ny-Ålesund District (altitude of 100 m) of Arctic (78°55′ N). Due to its chemical and morphological features as well as the 18S rDNA (GenBank accession number FJ430580, 100% similarity), the strain can be assigned to the genus *Eutypella* sp. The fungus was deposited in the PDA medium at the China Center for Type Culture Collection, Wuhan, China. The mycelium of the strain was picked and inoculated in a 50 mL flask filled with PDB (24 g/L of potato dextrose broth) medium, and cultured at 28 °C with 180 rpm for 3 days to obtain the seed culture.

### 3.3. Fermentation

The seed was added to 500 mL of PDB medium in a ratio of 5% (*V*/*V*), and the medium was placed in a shaker at 180 rpm under condition of 20 °C for 10 days. The ethanol was added at 72, 96, and 120 h of cultivation, respectively, with a concentration of 4%.

### 3.4. Extraction and Isolation

The fermentation broth was filtered using eight layers of gauze, and the filtrate was thoroughly mixed with an equal volume of ethyl acetate. The ethyl acetate layer was collected after layering, and this process was repeated three times. The ethyl acetate was evaporated using a rotary evaporator to obtain the fermentation products.

The crude extracts (58.8 g) were separated into 14 fractions (A–N) using middle-pressure liquid chromatography with MeOH/H_2_O (0–70 min, 10–50%; 71–310 min, and 50–100%). The fraction H was separated on an ODS (50 µm) column followed by stepwise gradient elution with MeOH/H_2_O (50–100%) to obtain five subfractions (H1–H9). The fraction H6 was subjected to vacuum liquid chromatography on silica gel eluting with a step gradient of a mixture of petroleum ether and EtOAc (from 50:1 to 0:1) to afford seven fractions. Fractions were purified by HPLC (MeOH/H_2_O, 2.0 mL/min) detected at the wavelength of 210 nm to afford compound **1** (1.7 mg), compound **2** (6.2 mg), and compound **3** (2.1 mg). The separation process of known compounds is not elaborated.

Eutypellenone F (**1**): white powder; [*α*${]}_{D}^{25}$-90.35 (*c* 0.05, MeOH); UV (MeOH) *λ*_max_ (log *ε*) 206 (4.24) nm; CD (MeOH) (∆*ε*) 205 (−1.99), 235 (−0.89), 285 (−0.78); ^1^H-NMR and ^13^C-NMR data, see Table 1; and HRESIMS *m/z* 483.2348 [M+Na]^+^ (calcd for C_26_H_36_O_7_Na, 483.2353).

Libertellenone Y (**2**): yellow oil; [*α*${]}_{D}^{25}$-15.8 (*c* 0.05, MeOH); UV (MeOH) *λ*max (log *ε*) 205 (3.94), 250 (3.73), 311 (3.26) nm; CD (MeOH) (Δ*ε*) 210 (+8.28), 237 (+2.99), 278 (−1.67), 351 (+1.34) nm; ^1^H-NMR and ^13^C-NMR data, see Table 1; and HRESIMS *m/z* 511.2310 [M+Na]^+^ (calcd for C_27_H_36_O_8_Na, 511.2302).

Libertellenone Z (**3**): yellow oil; [*α*${]}_{D}^{25}$-78.63 (*c* 0.05, MeOH); UV (MeOH) *λ*_max_ (log *ε*) 266 (4.14) nm, 324 (4.34) nm; CD (MeOH) (∆*ε*) 204 (+1.93), 220 (−1.44), 275 (+0.82), 312 (+0.64), 352 (−2.56); ^1^H-NMR and ^13^C-NMR data, see Table 1; and HRESIMS *m/z* 355.1880 [M+Na]^+^ (calcd for C_20_H_28_O_4_Na, 355.1879).

### 3.5. ECD Calculations

The conformational analyses for compounds **1**–**3** were initially performed using Spartan’10 software (Wave-function, Inc., Irvine, CA, USA) in the MMFF94 force field. Subsequently, the conformers with a Boltzmann population of over 5% were optimized at the B3LYP/6-31+G (d, p) level by employing the conductor-like polarizable continuum model (CPCM) in MeOH. The theoretical calculation of ECD for **1**–**3** was calculated using the time-dependent density functional theory (TDDFT) methodology at the B3LYP/6-311++G (2d, 2p) level in MeOH, respectively. The ECD spectra were generated by the program SpecDis 1.6 (University of Würzburg, Würzburg, Germany) using a Gaussian band shape with 0.3 eV exponential half-width from dipole-length dipolar and rotational strengths.

### 3.6. Assay of Anti-Inflammatory Activity Based on LPS-Induced RAW264.7 Model

Mouse macrophage RAW264.7 was diluted with DMEM medium containing 10% serum to 2 × 10^5^ cells/mL into 96 well plate, and cultured in 37 °C for 24 h. The compounds **1**–**7** were dissolved in DMSO and prepared in different concentrations using DMEM medium to add to the 96-well plate. CCK-8 reagent was added to the 96-well plate, and the cells were cultured for three hours. The absorbance of medium was measured at 450 nm to obtain the cell survival rate. LPS (TLR4 activator) was added to the 96-well plate with a concentration of 1 μg/mL, and the cells were cultured in 37 °C for 24 h. The absorbance of supernatant was measured at 540 nm to detect the content of NO. The formula for calculating the inhibition rate of NO release is: inhibition rate = (LPS group NO content—compound group NO content)/LPS group NO content × 100%.

### 3.7. Assay of Anti-Inflammatory Activity Based on Zebrafish Model

The transgenic zebrafish *Tg (zlyz:EGFP)* provided by Engineering Research Center of Zebrafish Models for Human Diseases and Drug Screening of Shandong Province was selected with normal development. The zebrafishes were transferred into a 24-well plate, with 12 fishes per well. The blank control group, copper sulfate model group, and compounds group (10 μmol/L, 20 μmol/L, and 40 μmol/L) were set up, and each concentration group was equipped with three repeated wells. Copper sulfate was added to all groups except the blank group after 24 h of culturing, with a concentration of 40 μmol/L. The migration of inflammatory cells was observed under a fluorescence microscope after two hours of culturing, and the number of inflammatory cells migrating to the lateral line behind the cloaca was calculated.

### 3.8. Statistical Analysis

All statistical analyses were conducted using SPSS 10.0 software. Data are expressed as the mean ± standard deviation (SD). Statistical analysis was performed using Student’s *t*-test. All the data were generated from at least three independent experiments; *p*-value of <0.05 was considered statistically significant.

## 4. Conclusions

In summary, the use of the OSMAC strategy can effectively change the secondary metabolites of polar fungus *Eutypella* sp. D-1 [21]. In this study, seven diterpenes of pimarane-type were obtained from strain fermentation broth by adding ethanol as a promoter in the medium, including three new compounds eutypellenone F, libertellenone Y, and libertellenone Z, and libertellenone Y possess a rare tetrahydrofuran-fused pimarane diterpene skeleton. The anti-inflammatory activity of all compounds was evaluated using the RAW264.7 inflammation model and zebrafish model, and the results showed that compounds **3**–**6** possess significant anti-inflammatory activity. This work indicated that the adding of a precursor in the medium leads to the production of new secondary metabolites with biological activity in the fungus.

## Figures and Tables

**Table 1:** ^1^H and ^13^C-NMR data of compounds **1**–**3** in CDCl_3_.

Position	1 *		2 *		3 *	
	*δ*c, type	*δ*_H_, mult. (*J* in Hz)	*δ*c, type	*δ*_H_, mult. (*J* in Hz)	*δ*c, type	*δ*_H_, mult. (*J* in Hz)
1a	38.46, CH	3.30, m	32.5, CH	3.26, m	30.58, CH_2_	1.95, m
1b						1.52, m
2a	25.62, CH_2_	2.28, m	27.0, CH_2_	2.34, m	17.34, CH_2_	1.69, m
2b		2.22, dd, (12.62, 4.95)		1.51, m		1.65, m
3a	74.90, CH	5.21, m	73.0, CH	5.12, d, (1.1)	36.36, CH_2_	1.91, m
3b						1.36, m
4	41.51, C		40.6, C		41.32, C	
5	125.88, C		126.5, C		138.79, C	
6	153.37, C		179.8, C		143. 82, C	
7	115.25, CH	6.86, s	145.4, C		181.80, C	
8	137.12, C		122.3, C		133.71, C	
9	75.42, CH	4.29, m	78.0, C		147. 97, CH	7.04, d, (1.86)
10	40.13, C		167.2, C		38.73, C	
11a	31.62, CH_2_	1.96, m	35.1, CH_2_	2.04, m	29.36, CH_2_	1.84, m
11b		1.77, m		1.50, m		1.57, m
12a	22.64, CH_2_	2.74, m	28.2, CH_2_	1.52, m	25.66, CH_2_	1.92, m
12b				2.18, m		1.80, m
13	125.84, C		44.3, C		74.86, C	
14	138.70, C		80.1, CH	4.40, s	44.45, C	
14-OCH_3_			57.5, OCH_3_	3.34, s		
15a	63.96, CH_2_	3.73, m	143.2, CH	5.69, dd, (17.8, 11.1)	70.49, CH_2_	4.48, d, (8.10)
15b						3.16, d, (11.36)
16a	21.45, CH_3_	2.17, s	113.6, CH_2_	5.01, dd, (11.2, 1.0)	22.38, CH_3_	1.17, s
16b				5.08, d, (1.0)		
17	171.50, C		25.5, CH_3_	1.20, s	29.73, CH_3_	1.20, s
18a	18.78, CH_3_	0.90, d, (7.51)	65.2, CH_2_	4.35, d, (10.5)	23.17, CH_3_	1.12, s
18b				4.79, d, (10.5)		
19	18.64, CH_3_	0.98, d, (7.51)	21.0, CH_3_	1.36, s	145.36, CH	5.88, q, (6.41)
20a	34.06, CH	2.37, m	72.3, CH_2_	4.42, t, (7.95)	112.63, CH_2_	5.08, m
20b				3.75, dd, (8.7, 7.4)		
21	176.80, C		170.1, C			
22a	65.42, CH_2_	5.0, d, (11.19)	21.1, CH_3_	2.0, s		
22b		4.30, m				
23	21.81, CH_3_	1.54, s	176.6, C			
24	23.06, CH_3_	1.18, s	34.3, CH	2.53, m		
25	140.86, CH	5.83, q, (7.24)	19.1, CH_3_	1.13, d, (7.0)		
26	116.44, CH_2_	5.21, m	19.1, CH_3_	1.14, d, (7.0)		

* 500 MHz for ^1^H-NMR and 125 MHz for ^13^C-NMR.

**Table 2:** The NO release inhibition rate of compounds.

Compound	Inhibition (%)
**1**	<20
**2**	<20
**3**	60.93
**4**	89.4
**5**	84.2
**6**	23.17
**7**	<20
Dexamethasone	72.85

## Data Availability

The data presented in this study are available on request from the corresponding author.

## Supplementary Materials

The following supporting information can be downloaded at: https://www.mdpi.com/article/10.3390/md21100541/s1. Figures S1–S36 spectrums of compounds **1**–**7.** Figure S37 The colony and mycelium characteristics of *Eutypella* sp. D-1
