# Supplementary material for: Pimarane-Type Diterpenes with Anti-Inflammatory Activity from Arctic-Derived Fungus Eutypella sp. D-1
Source: Mar Drugs. 2023 Oct 18;21(10):541. doi: 10.3390/md21100541 (PMC10608715; doi:10.3390/md21100541)

Supplementary data

Pimarane-type diterpenes with anti-inflammatory activity from Arctic-derived fungus *Eutypella* sp. D-1

Yaodong Ning [1,†], Shi Zhang [2,†], Te Zheng [3], Yao Xu [1], Song Li [1], Jianpeng Zhang [1], Binghua Jiao [1], Yun Zhang [3,*], Zengling Ma [2,*] and Xiaoling Lu [1,*]

[1] Department of Biochemistry and Molecular Biology, College of Basic Medical Sciences, Naval Medical University, Shanghai 200433, China
[2] College of Life and Environmental Sciences, Wenzhou University, Wenzhou 325000, China
[3] Biology Institute, Qilu University of Technology (Shandong Academy of Sciences), Jinan 250000, China

* Correspondence: luxiaoling80@126.com
† These authors contributed equally to this work.

S1. ^{1}H NMR spectrum of eutypellenone F (**1**) in $CDCl_3$.
S2. ^{13}C NMR spectrum of eutypellenone F (**1**) in $CDCl_3$.
S3. DEPT135 spectrum of eutypellenone F (**1**) in $CDCl_3$.
S4. HSQC spectrum of eutypellenone F (**1**) in $CDCl_3$.
S5. COSY spectrum of eutypellenone F (**1**) in $CDCl_3$.
S6. HMBC spectrum of eutypellenone F (**1**) in $CDCl_3$.
S7. NOESY spectrum of eutypellenone F (**1**) in $CDCl_3$.
S8. HRESIMS of eutypellenone F (**1**).
S9. UV spectrum of eutypellenone F (**1**).
S10. ORD result of eutypellenone F (**1**)
S11. ^{1}H NMR spectrum of libertellenone Y (**2**) in $CDCl_3$.
S12. ^{13}C NMR spectrum of libertellenone Y (**2**) in $CDCl_3$.
S13. DEPT135 spectrum of libertellenone Y (**2**) in $CDCl_3$.
S14. HSQC spectrum of libertellenone Y (**2**) in $CDCl_3$.
S15. COSY spectrum of libertellenone Y (**2**) in $CDCl_3$.
S16. HMBC spectrum of libertellenone Y (**2**) in $CDCl_3$.
S17. NOESY spectrum of libertellenone Y (**2**) in $CDCl_3$.
S18. HRESIMS of libertellenone Y (**2**).
S19. ^{1}H NMR spectrum of libertellenone Z (**3**) in $CDCl_3$.
S20. ^{13}C NMR spectrum of libertellenone Z (**3**) in $CDCl_3$.
S21. DEPT135 spectrum of libertellenone Z (**3**) in $CDCl_3$.
S22. HSQC spectrum of libertellenone Z (**3**) in $CDCl_3$.
S23. COSY spectrum of libertellenone Z (**3**) in $CDCl_3$.
S24. HMBC spectrum of libertellenone Z (**3**) in $CDCl_3$.

S25. NOESY spectrum of libertellenone Z (**3**) in $CDCl_3$.
S26. HRESIMS of libertellenone Z (**3**).
S27. UV spectrum of libertellenone Z (**3**).
S28. ORD result of libertellenone Z (**3**)
S29. ^{1}H NMR spectrum of **4** in $CDCl_3$.
S30. ^{13}C NMR spectrum of **4** in $CDCl_3$.
S31. ^{1}H NMR spectrum of **5** in $CDCl_3$.
S32. ^{13}C NMR spectrum of **5** in $CDCl_3$.
S33. ^{1}H NMR spectrum of **6** in $CDCl_3$.
S34. ^{13}C NMR spectrum of **6** in $CDCl_3$.
S35. ^{1}H NMR spectrum of **7** in $CDCl_3$.
S36. ^{13}C NMR spectrum of **7** in $CDCl_3$.
S37. The colony and mycelium characteristics of *Eutypella* sp. D-1.

S1. ^{1}H NMR spectrum of eutypellenone F (**1**) in $CDCl_3$.

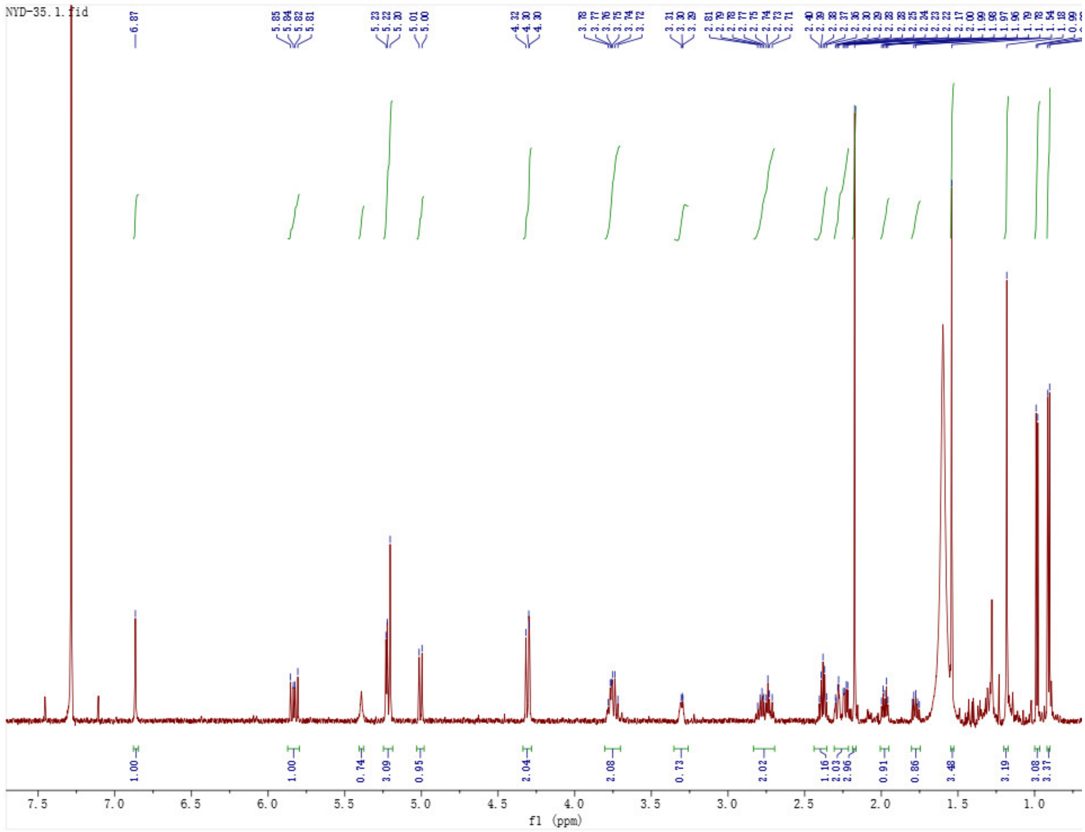

S2. ^{13}C NMR spectrum of eutypellenone F (**1**) in $CDCl_3$.

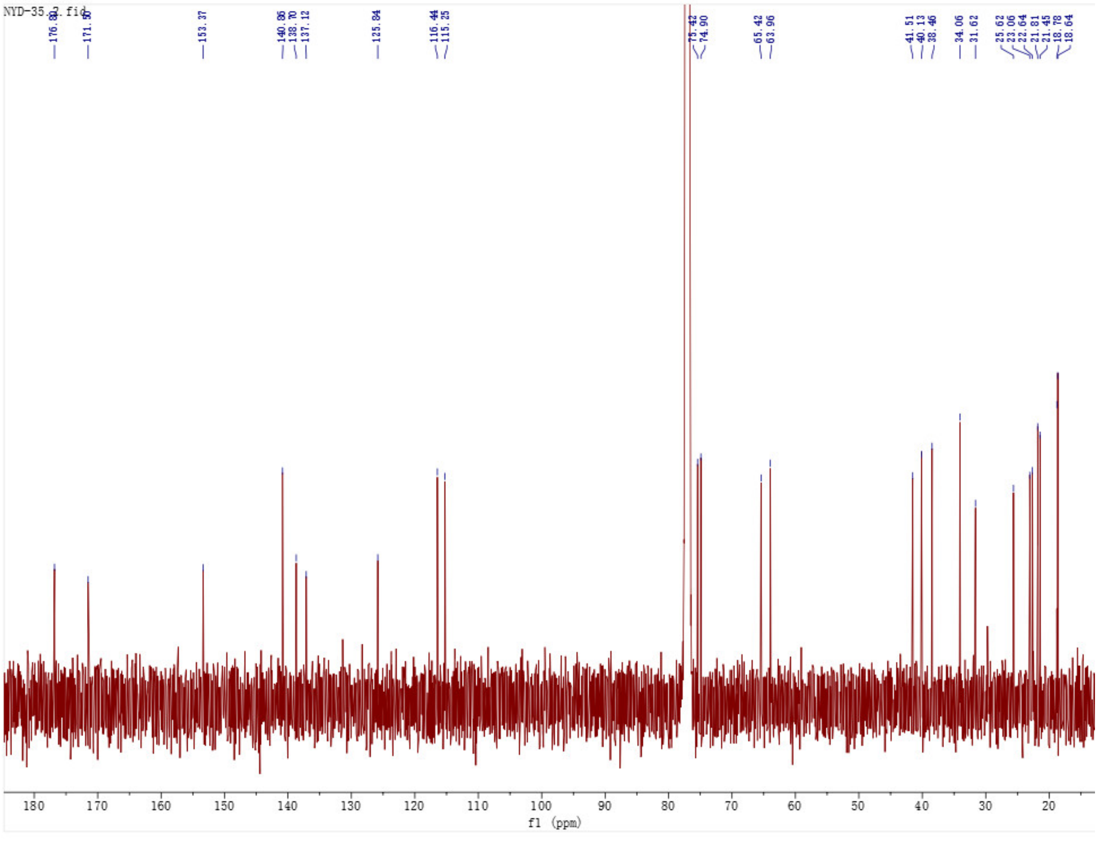

S3. DEPT135 spectrum of eutypellenone F (**1**) in $CDCl_3$.

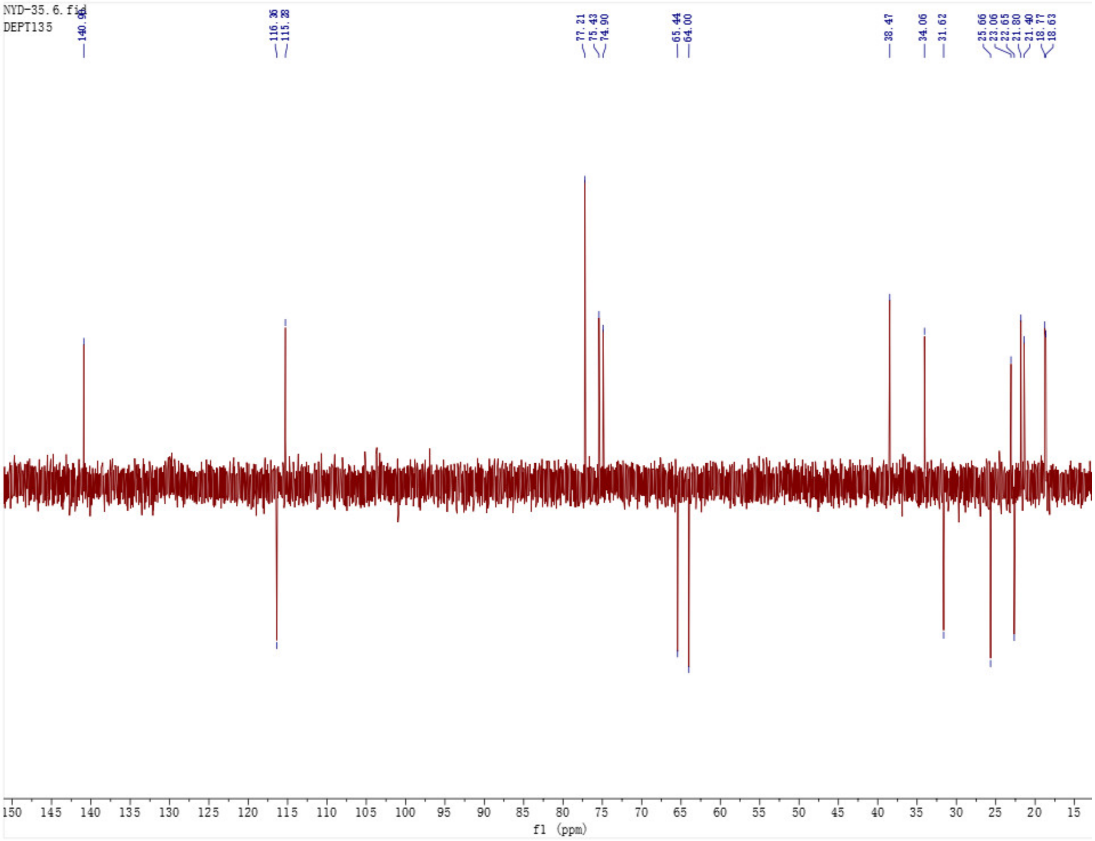

S4. HSQC spectrum of eutypellenone F (**1**) in $CDCl_3$.

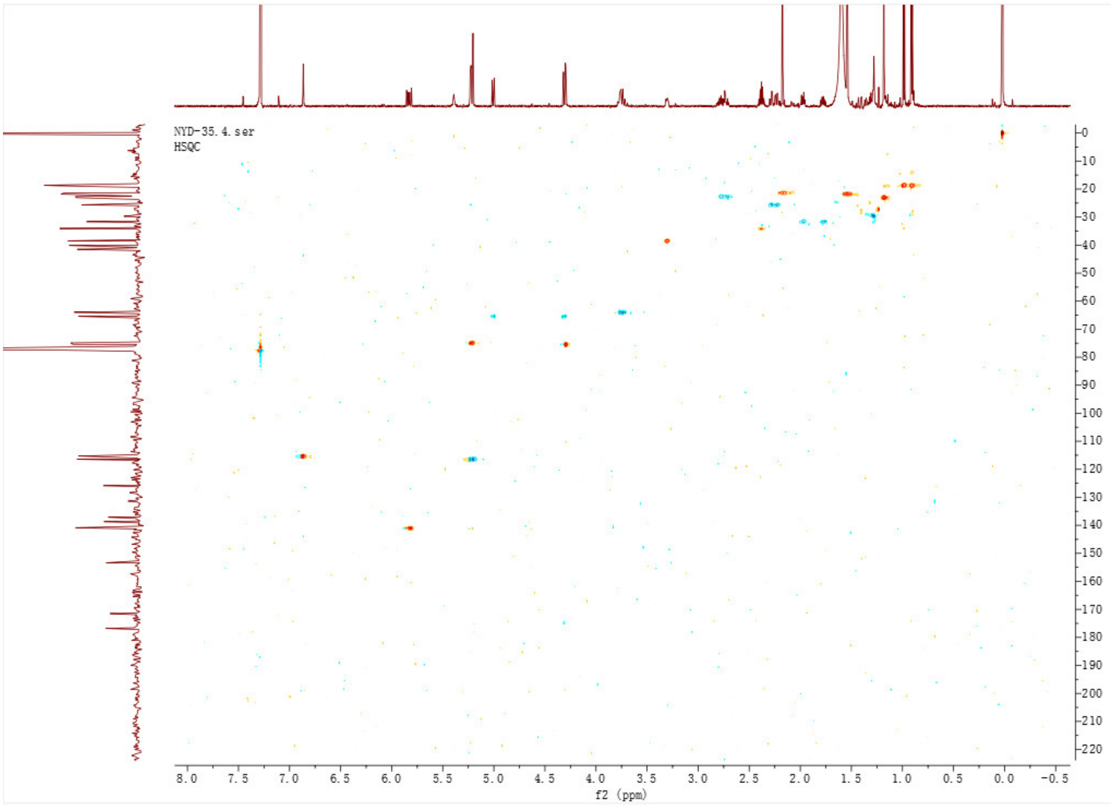

S5. COSY spectrum of eutypellenone F (**1**) in $CDCl_3$.

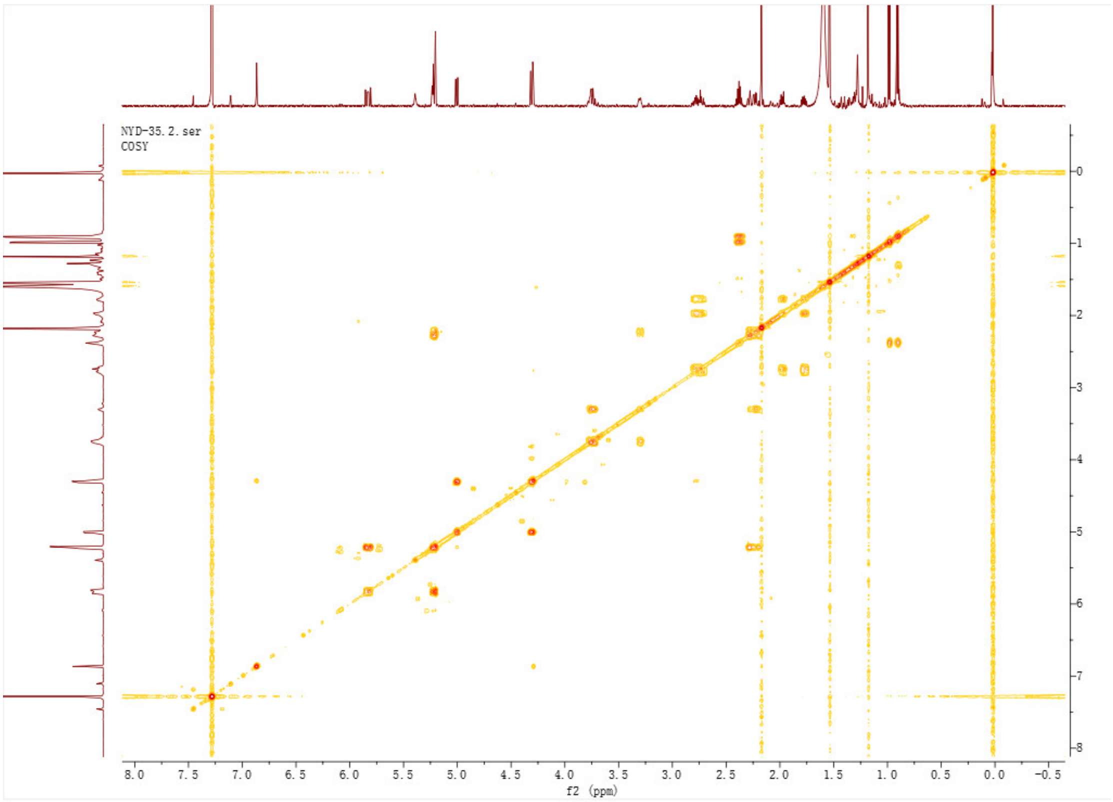

S6. HMBC spectrum of eutypellenone F (**1**) in $CDCl_3$.

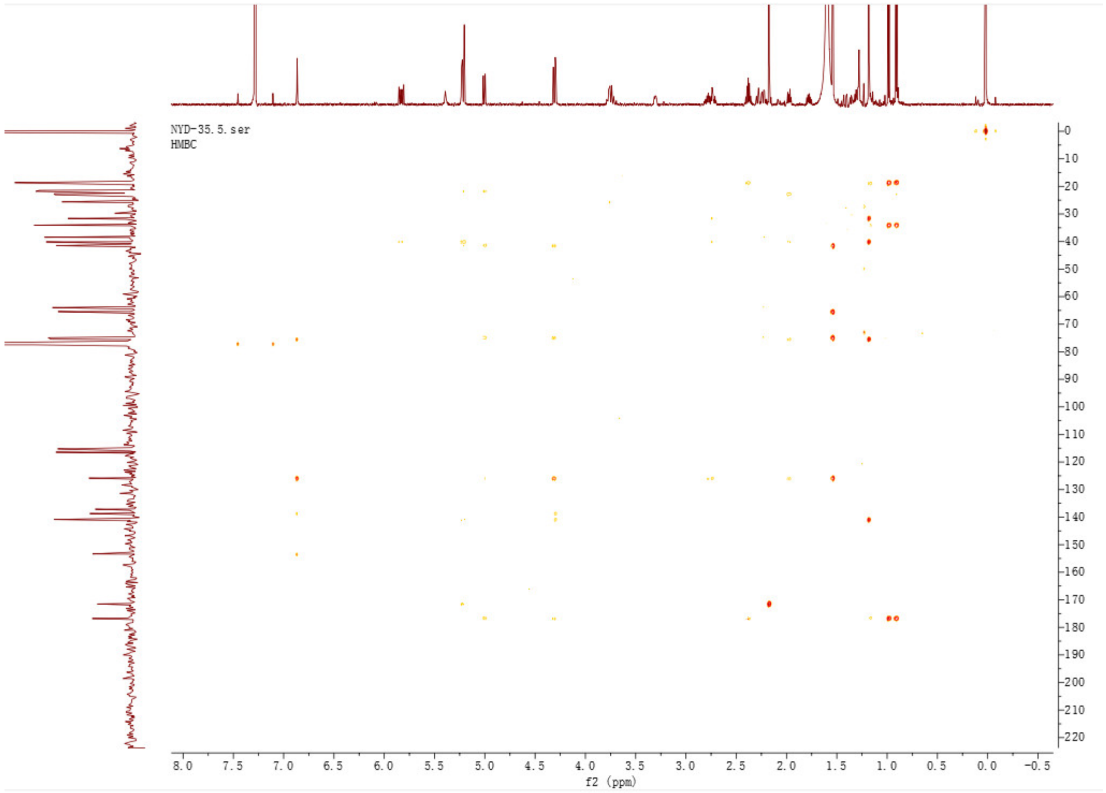

S7. NOESY spectrum of eutypellenone F (**1**) in $CDCl_3$.

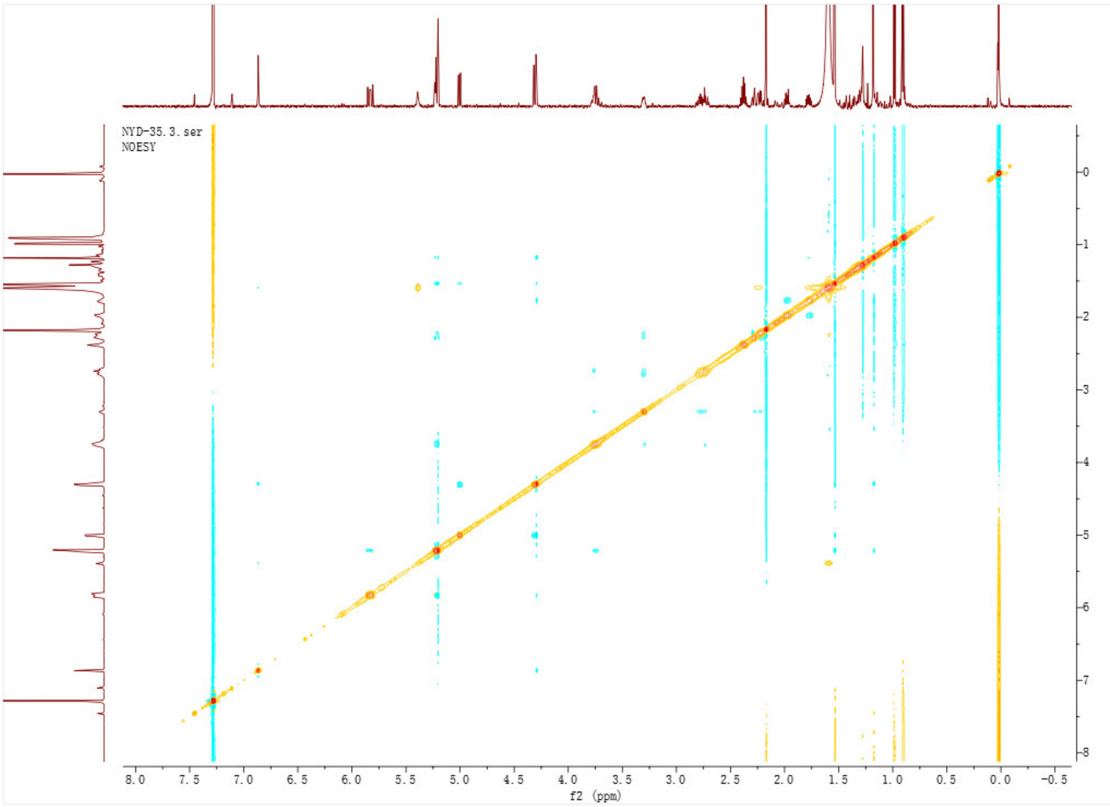

S8. HRESIMS of eutypellenone F (**1**).

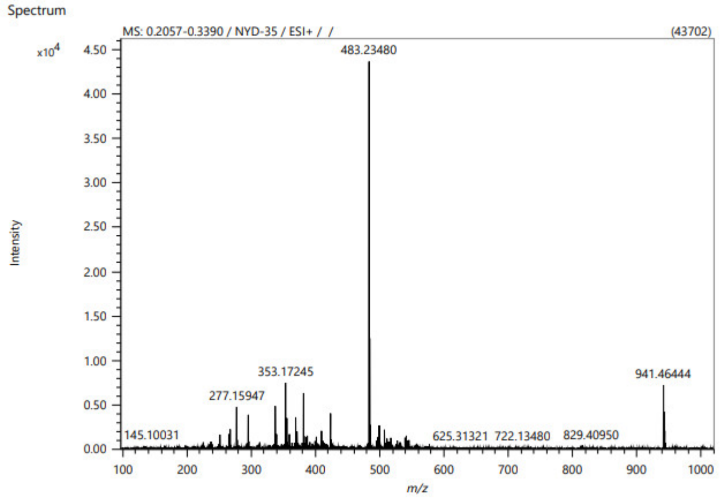

Elemental Composition

Parameters

Tolerance:	±5.00 ppm
Electron:	Odd/Even
Charge:	+1
DBE:	-1.5 - 200.0

Elements Set 1:

Symbol	C	H	N	O	Na	S	Cl	Br
Min	0	0	0	0	1	0	0	0
Max	200	200	0	8	1	0	0	0

Symbol	Si	F	B
Min	0	0	0
Max	0	0	0

Results

Mass	Intensity	Intensity [%]	Formula	Calculated Mass	Mass Difference [mDa]	Mass Difference [ppm]	DBE
483.23480	43702.00	100.00	C26 H36 O7 Na	483.23532	-0.52	-1.08	8.5

S9. UV spectrum of eutypellenone F (**1**).

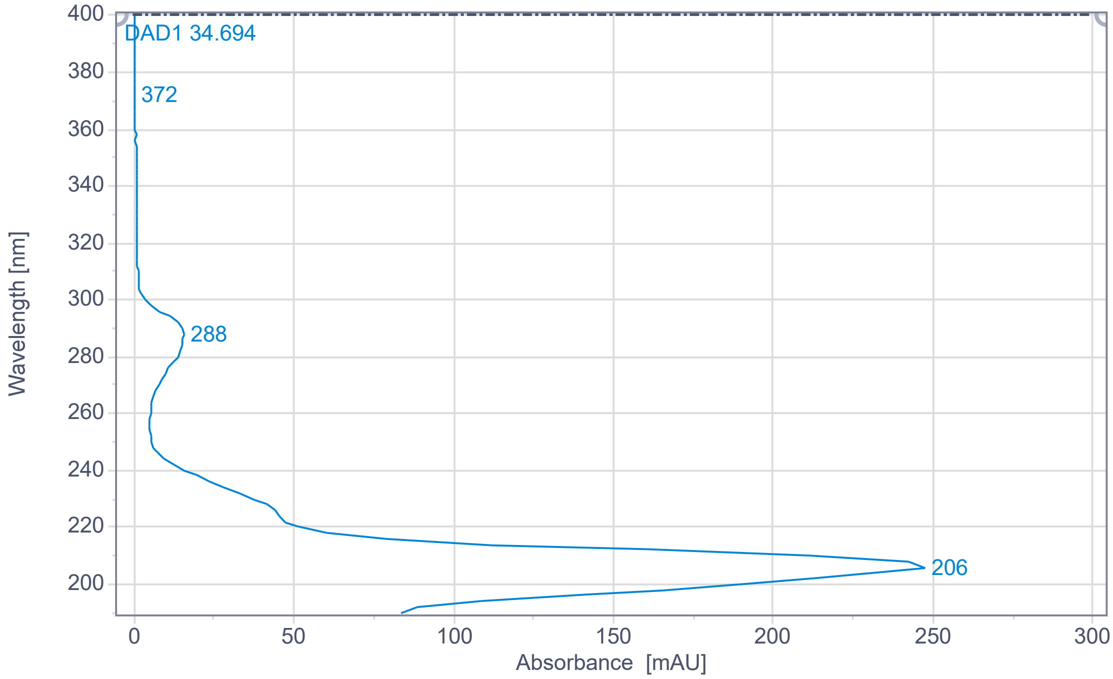

S10. ORD result of eutypellenone F (**1**)

Anton Paar MCP 5500 - Measurement Results:

Software version: 4.00.11383.92
MCP serial number: 99030100

Sample Information:

- Unique Sample Id: 51608
- Date: 2023-1-10
- Time: 15:24:32
- Method: Specific Rotation (25°C)
- Master Condition: valid
- Sample Name: NYD-35
- Concentration: 0.0500 g/100cm³
- User: student

Measurement Result:

Sub Measurement Number	Unique Sample Id	Time	Optical Rotation	Sample Cell Temperature	Specific Rotation (calc.)
			[°]	[°C]	[°]
1	51609	15:23:17	-0.0459	25.13	-91.7908
2	51610	15:23:35	-0.0451	25.06	-90.1910
3	51611	15:23:53	-0.0446	25.03	-89.1911
4	51612	15:24:12	-0.0451	25.00	-90.1910
5	51613	15:24:31	-0.0452	24.98	-90.3910
average	51608	15:24:32	-0.0452	25.04	-90.3510
std. dev.			0.000417	0.0525	0.833210

GxP Information (at 589 nm):

- Last Quartz Adjustment: 2021-11-30 15:41:39 by Administrator

S11. ^{1}H NMR spectrum of libertellenone Y (**2**) in $CDCl_3$.

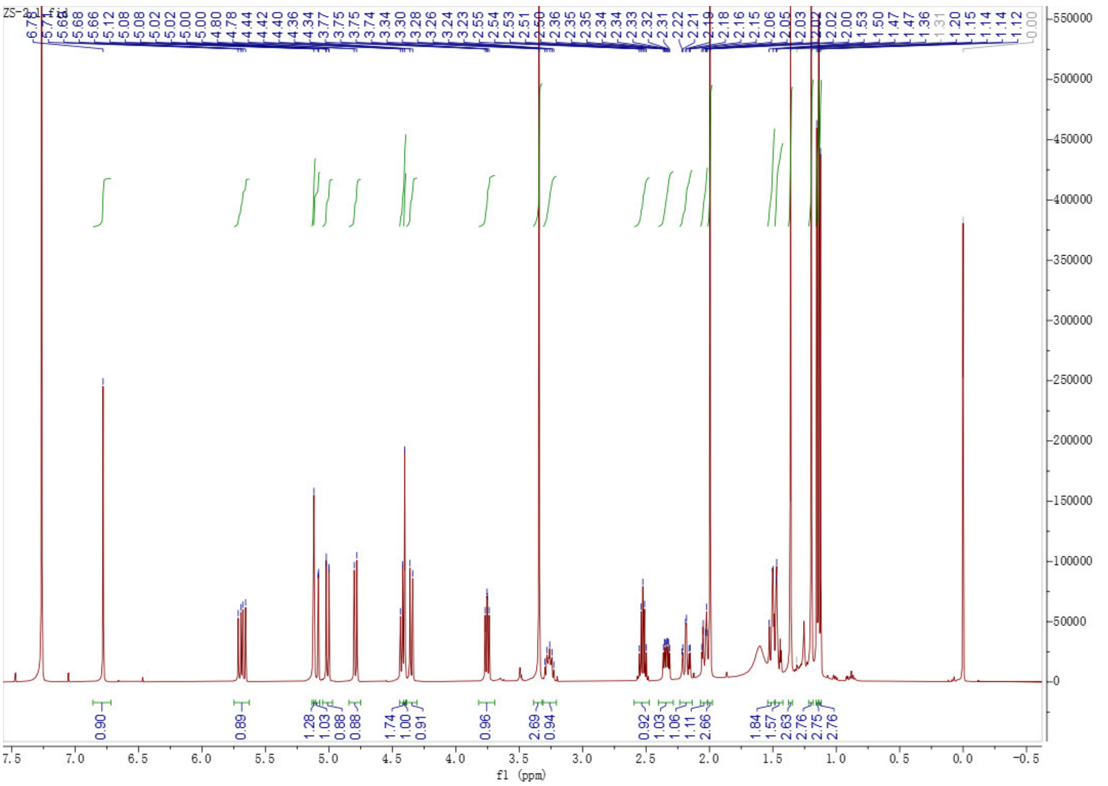

S12. ^{13}C NMR spectrum of libertellenone Y (**2**) in $CDCl_3$.

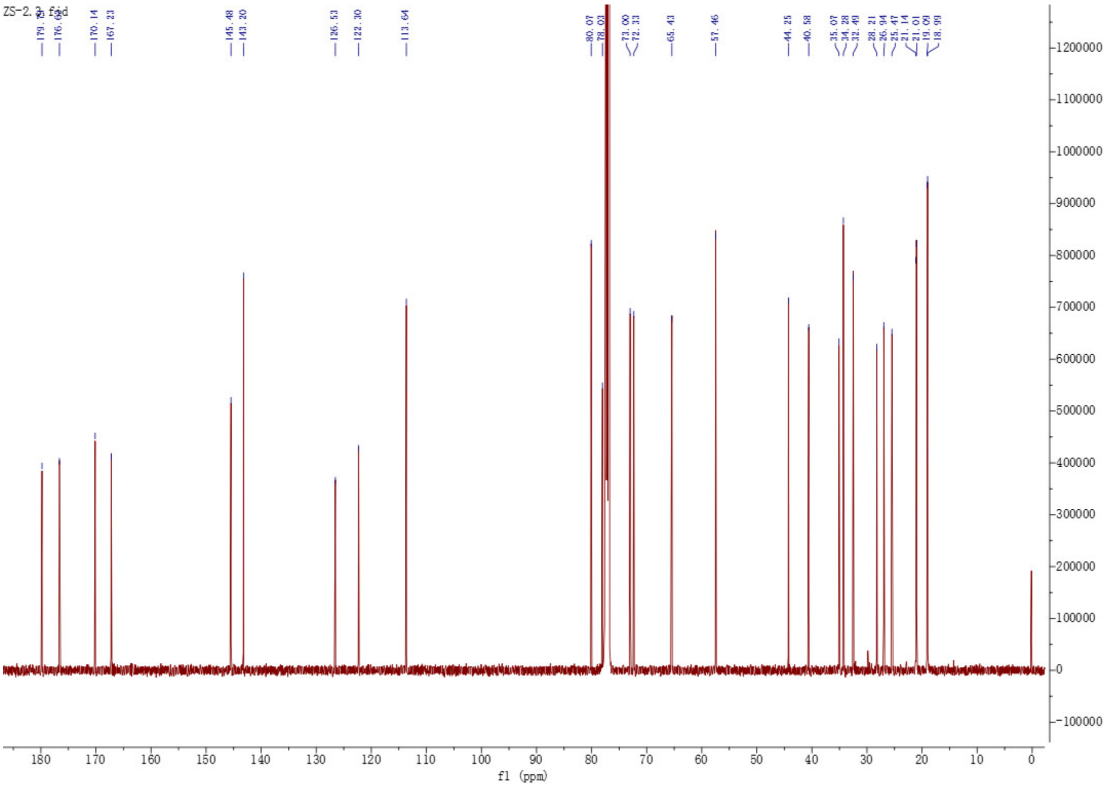

S13. DEPT135 spectrum of libertellenone Y (**2**) in $CDCl_3$.

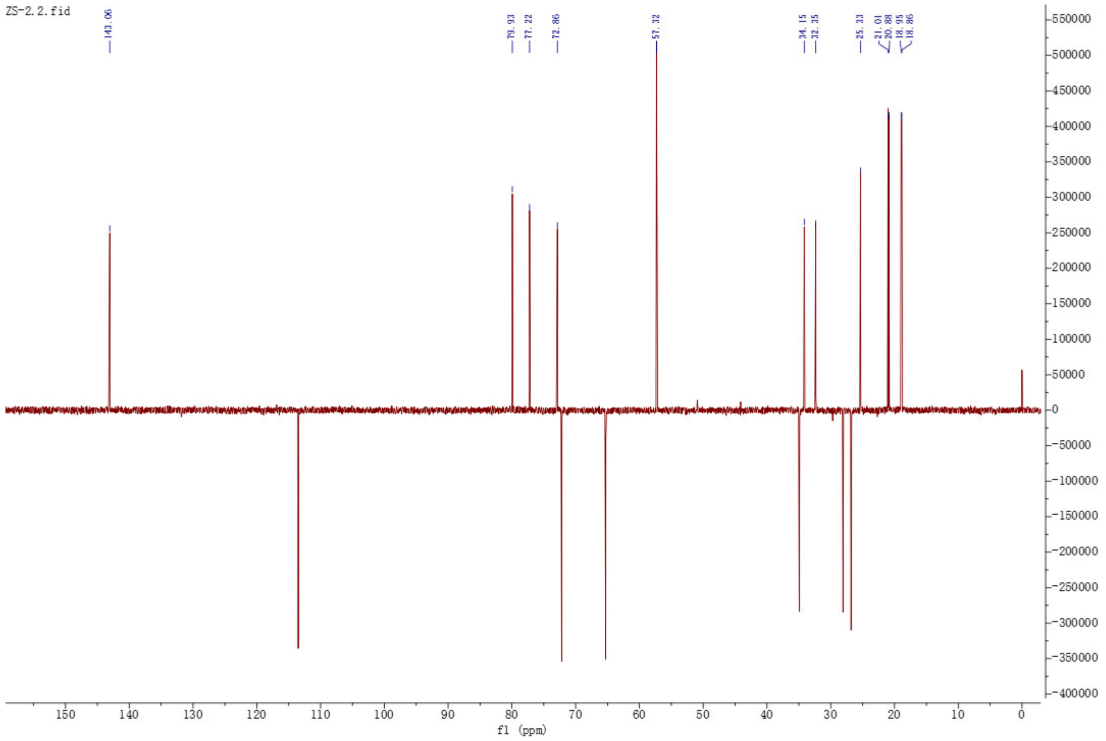

S14. HSQC spectrum of libertellenone Y (**2**) in $CDCl_3$.

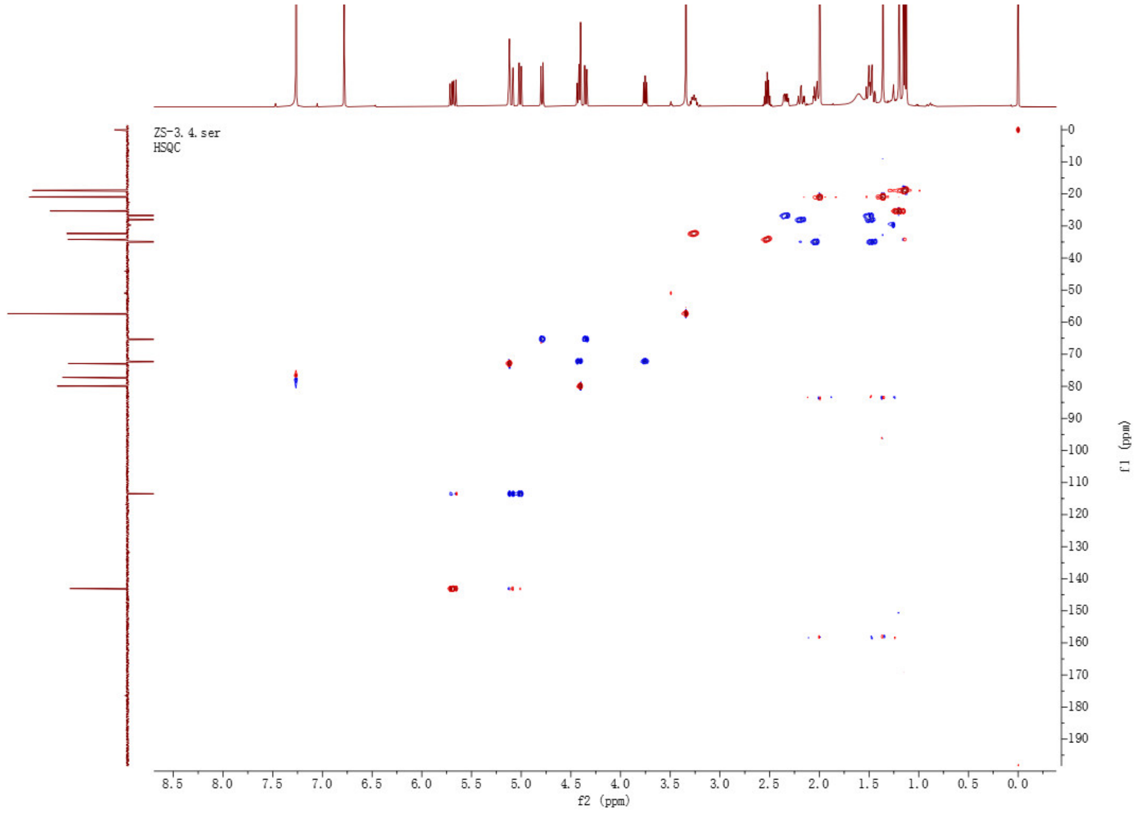

S15. COSY spectrum of libertellenone Y (**2**) in $CDCl_3$.

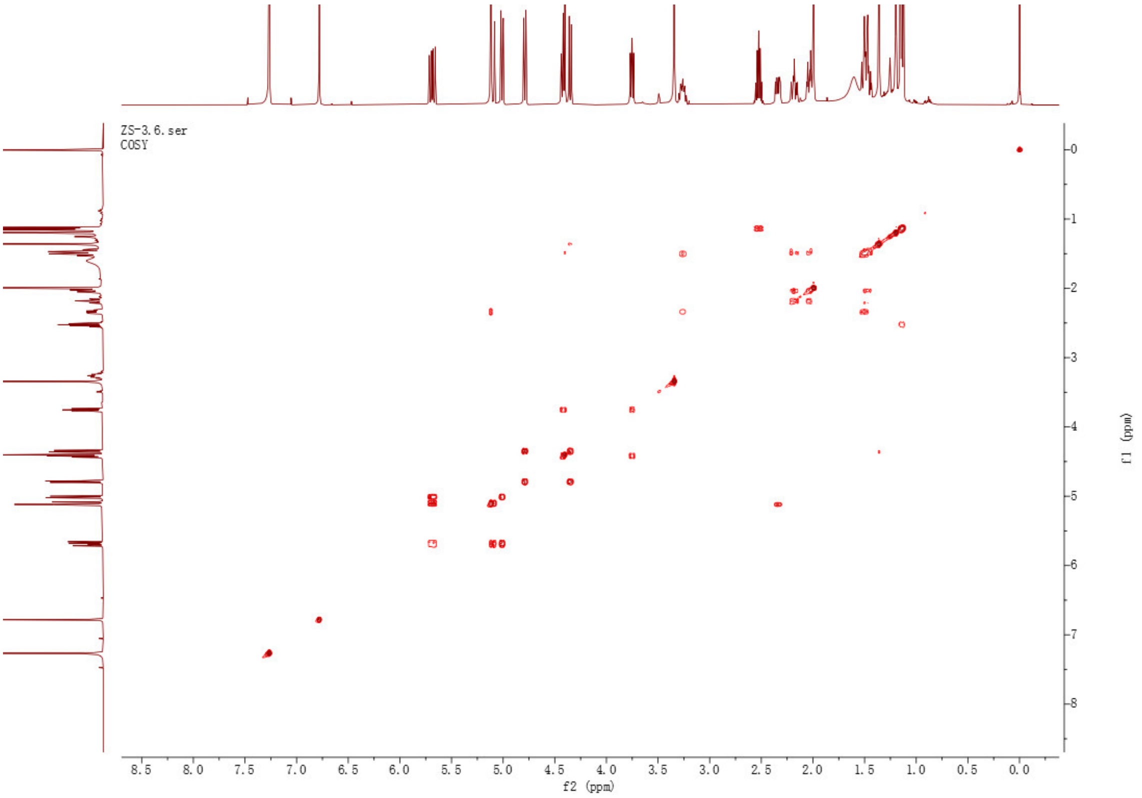

S16. HMBC spectrum of libertellenone Y (**2**) in $CDCl_3$.

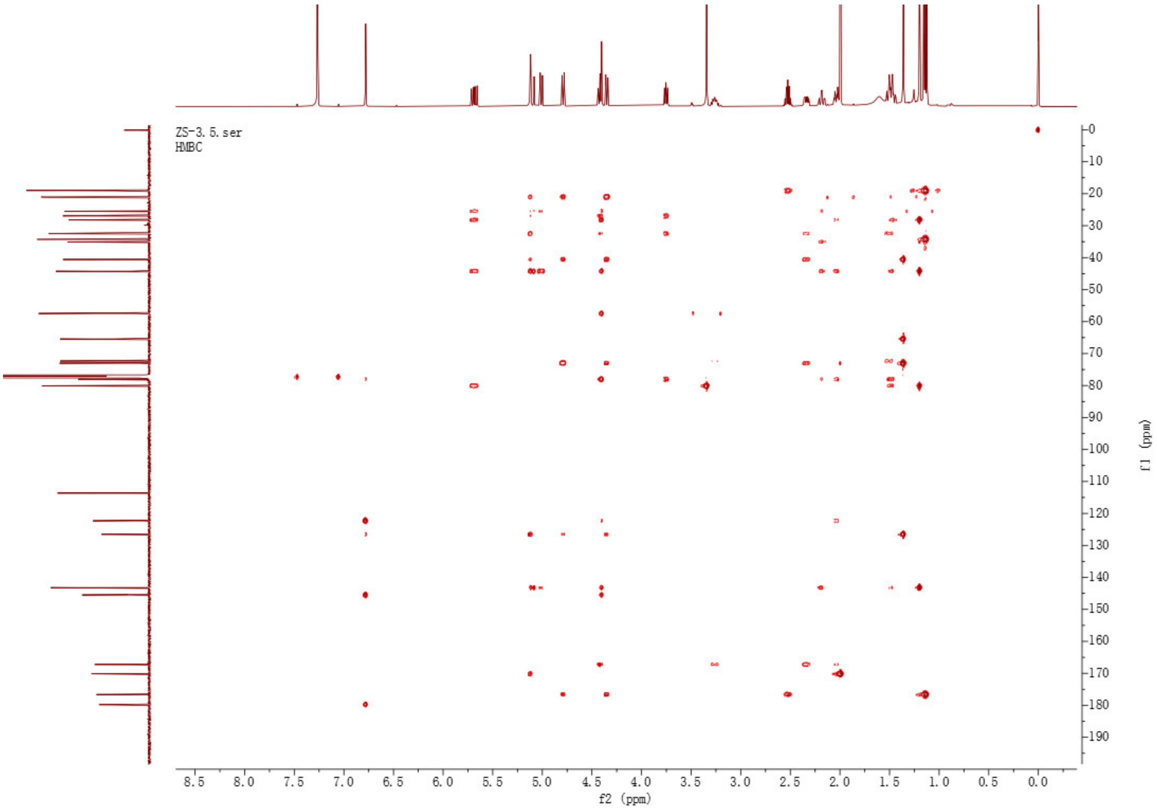

S17. NOESY spectrum of libertellenone Y (**2**) in $CDCl_3$.

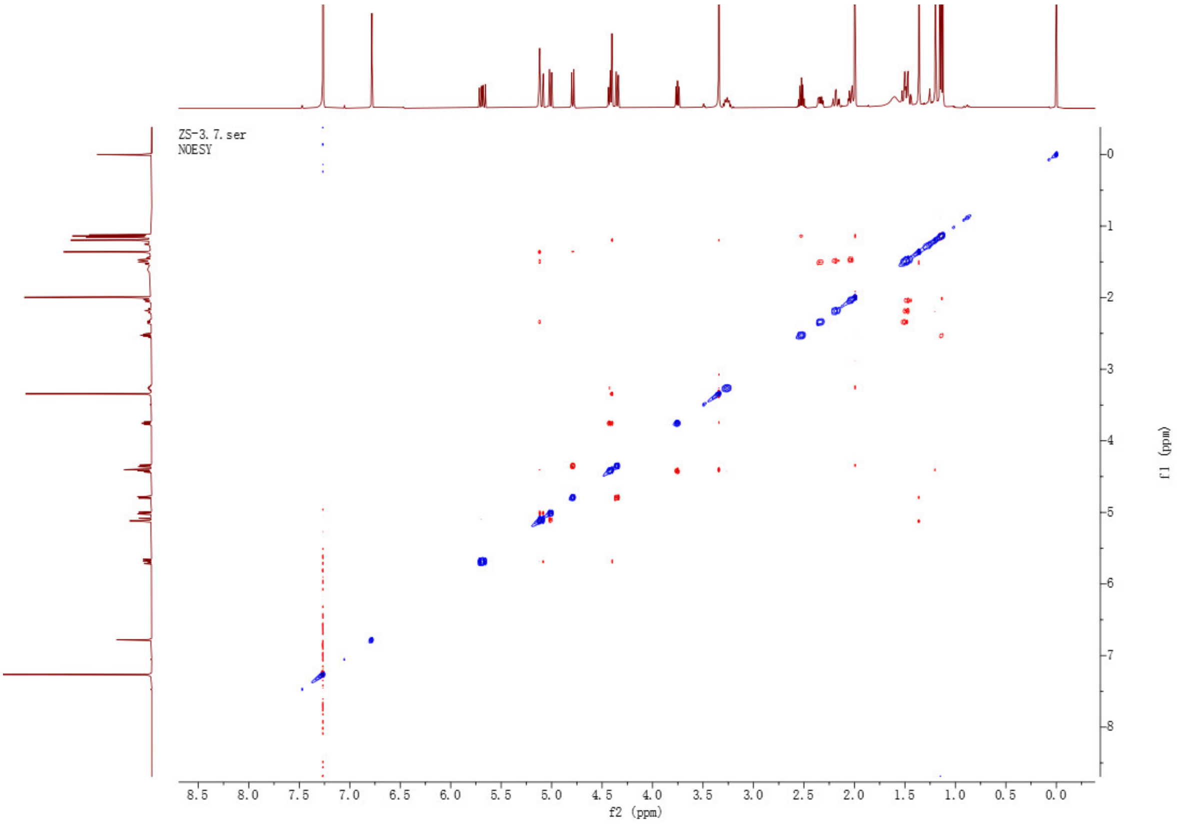

S18. HRESIMS of libertellenone Y (**2**).

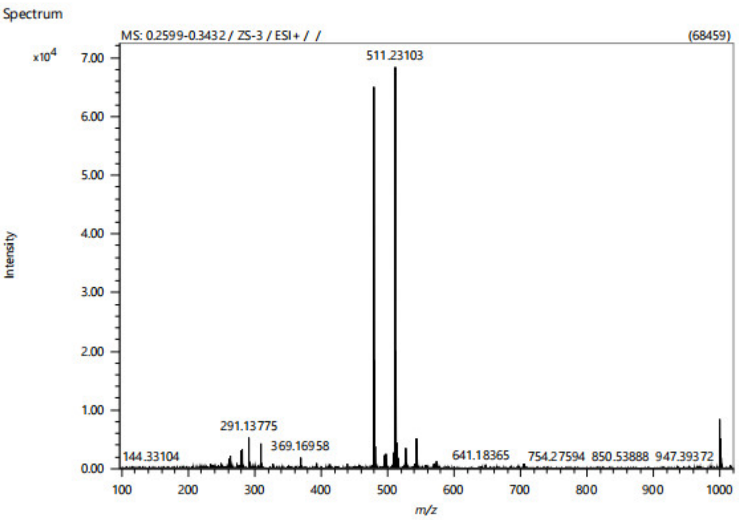

Elemental Composition

Parameters

Tolerance:	±5.00 ppm
Electron:	Odd/Even
Charge:	+1
DBE:	-1.5 - 200.0

Elements Set 1:

Symbol	C	H	N	O	Na	S	Cl	Br
Min	0	0	0	0	1	0	0	0
Max	200	200	0	8	1	0	0	0

Symbol	Si	F	B	P
Min	0	0	0	0
Max	0	0	0	0

Results

Mass	Intensity	Intensity [%]	Formula	Calculated Mass	Mass Difference [mDa]	Mass Difference [ppm]	DBE
511.23103	68459.15	100.00	C27 H36 O8 Na	511.23024	0.79	1.55	9.5

S19. ^{1}H NMR spectrum of libertellenone Z (**3**) in $CDCl_3$.

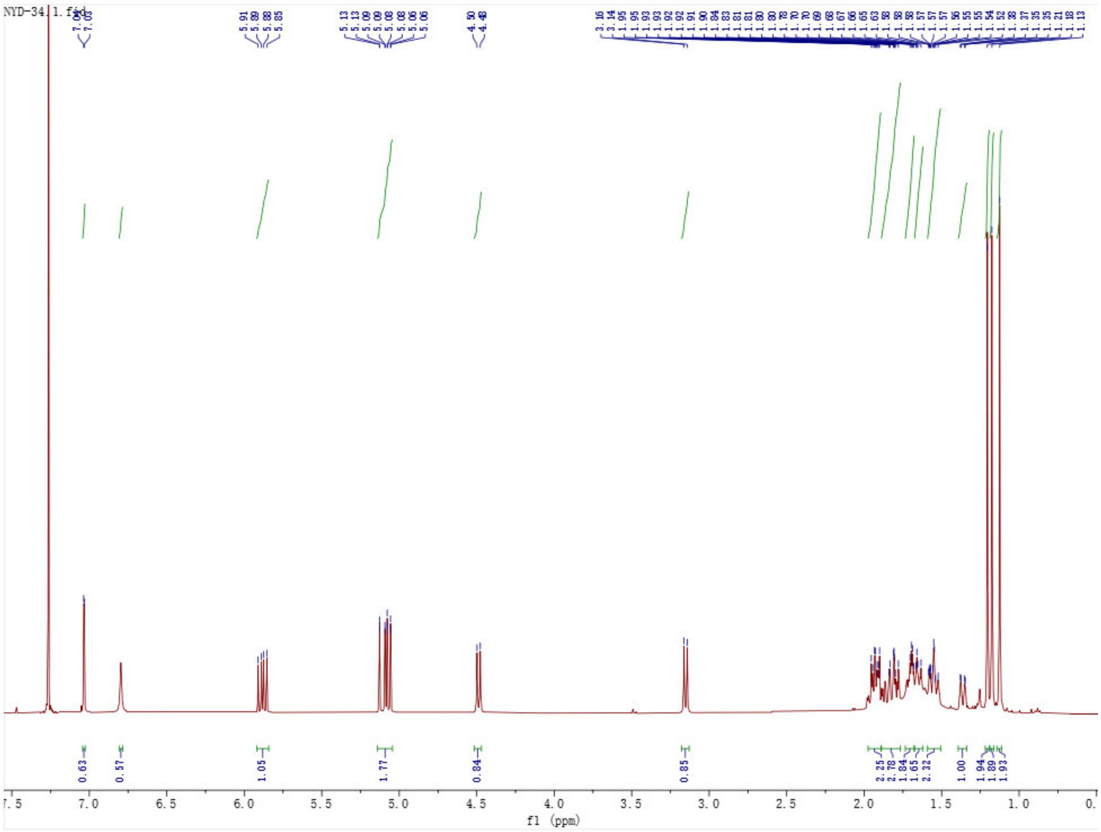

S20. ^{13}C NMR spectrum of libertellenone Z (**3**) in $CDCl_3$.

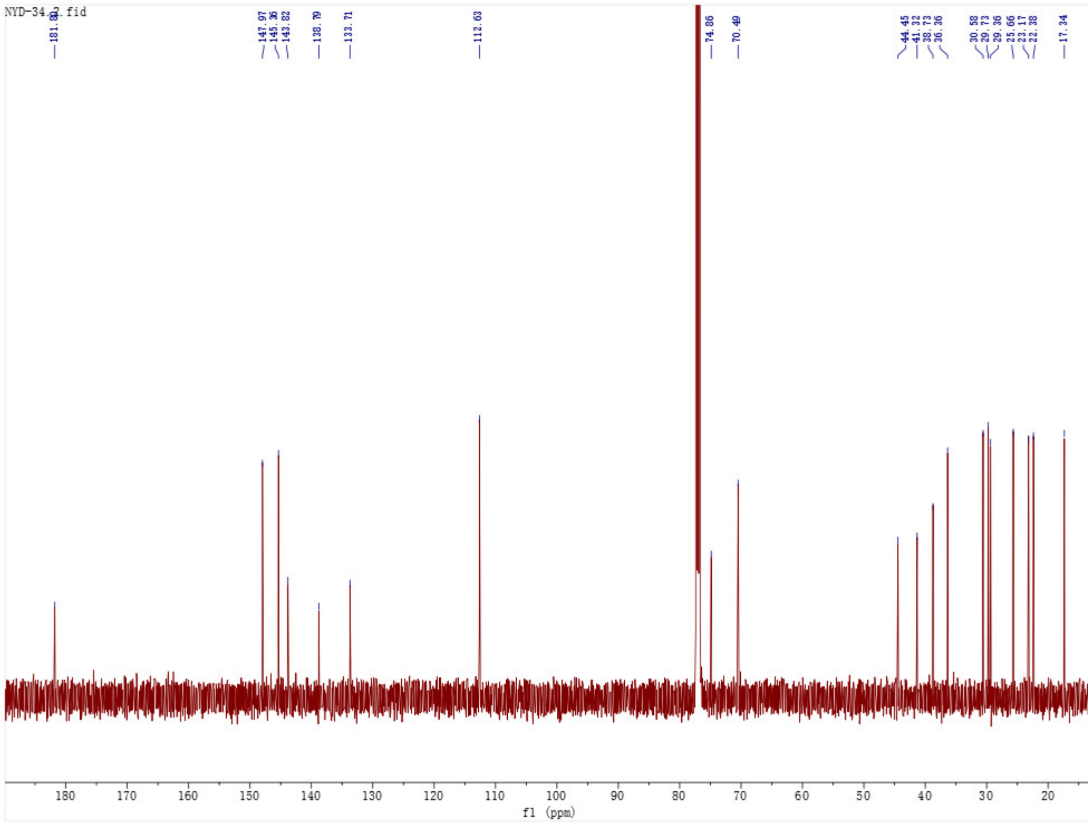

S21. DEPT135 spectrum of libertellenone Z (**3**) in $CDCl_3$.

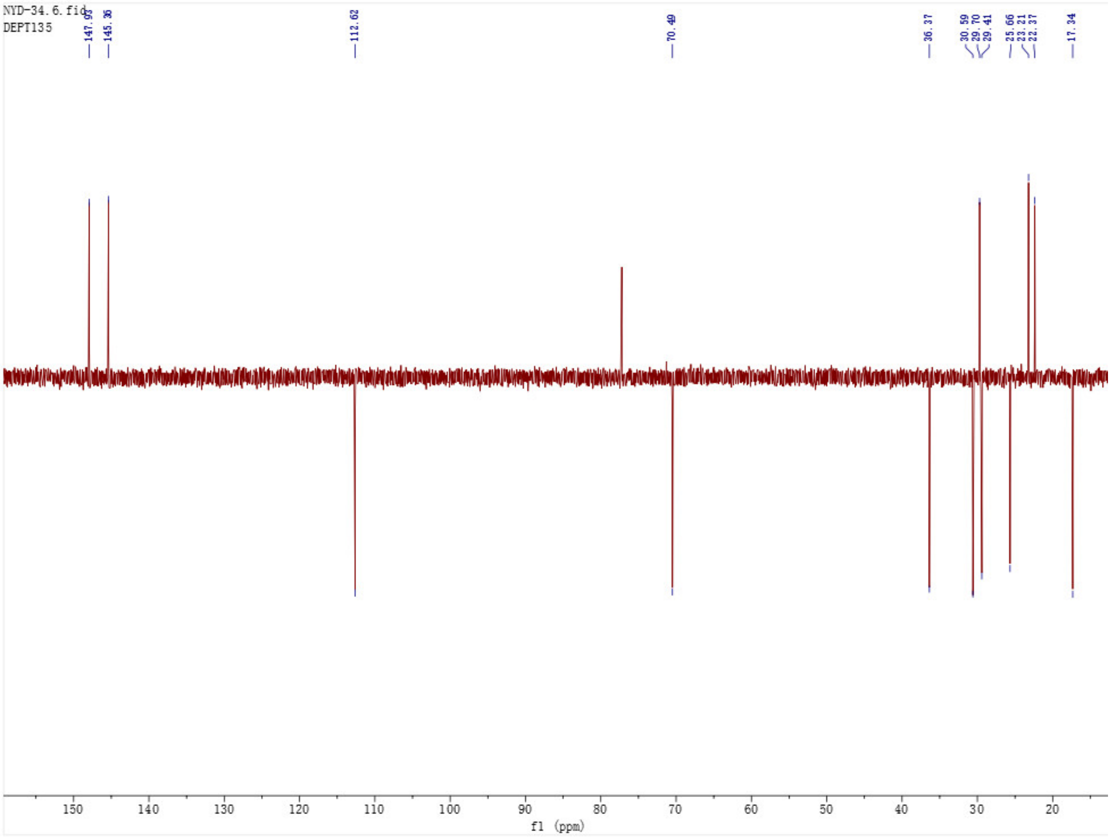

S22. HSQC spectrum of libertellenone Z (**3**) in $CDCl_3$.

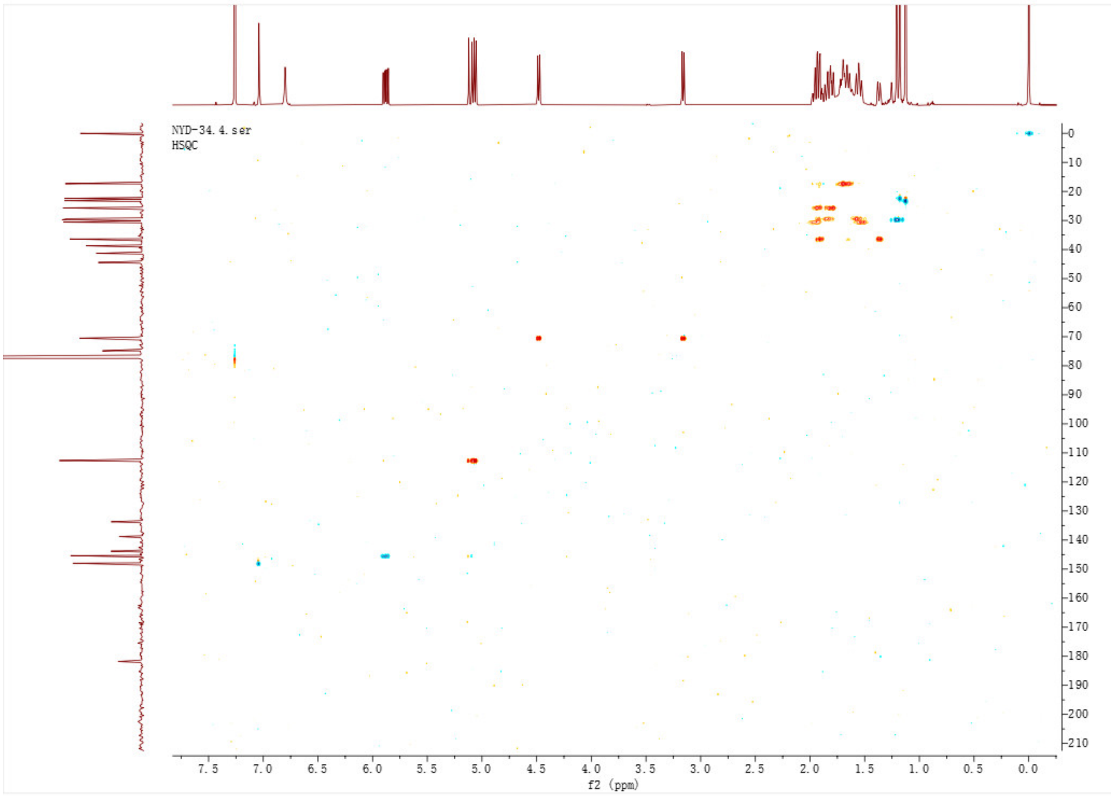

S23. COSY spectrum of libertellenone Z (**3**) in $CDCl_3$.

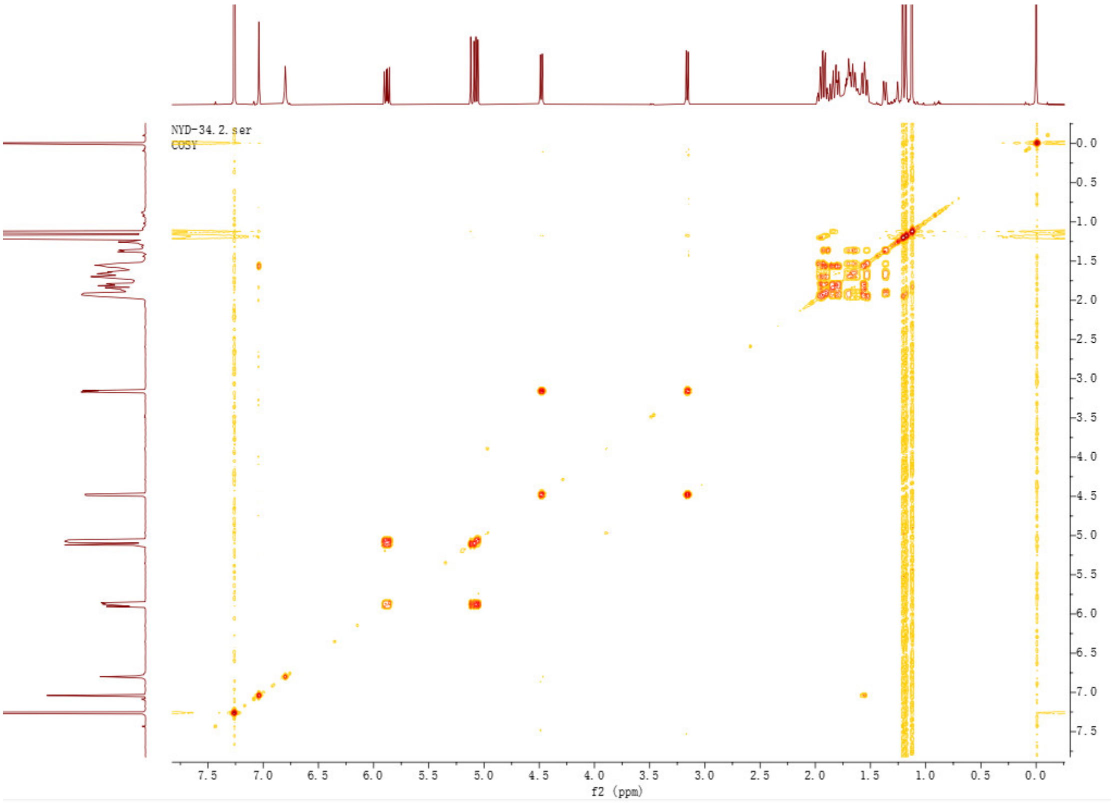

S24. HMBC spectrum of libertellenone Z (**3**) in $CDCl_3$.

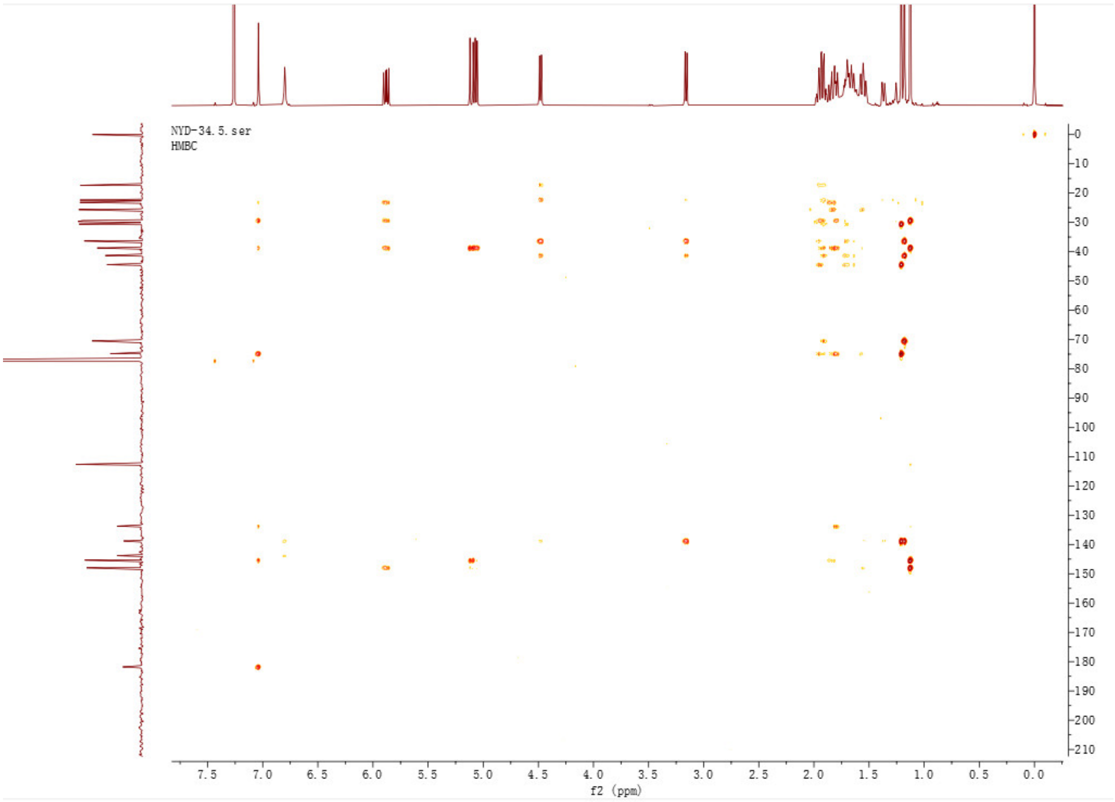

S25. NOESY spectrum of libertellenone Z (**3**) in $CDCl_3$.

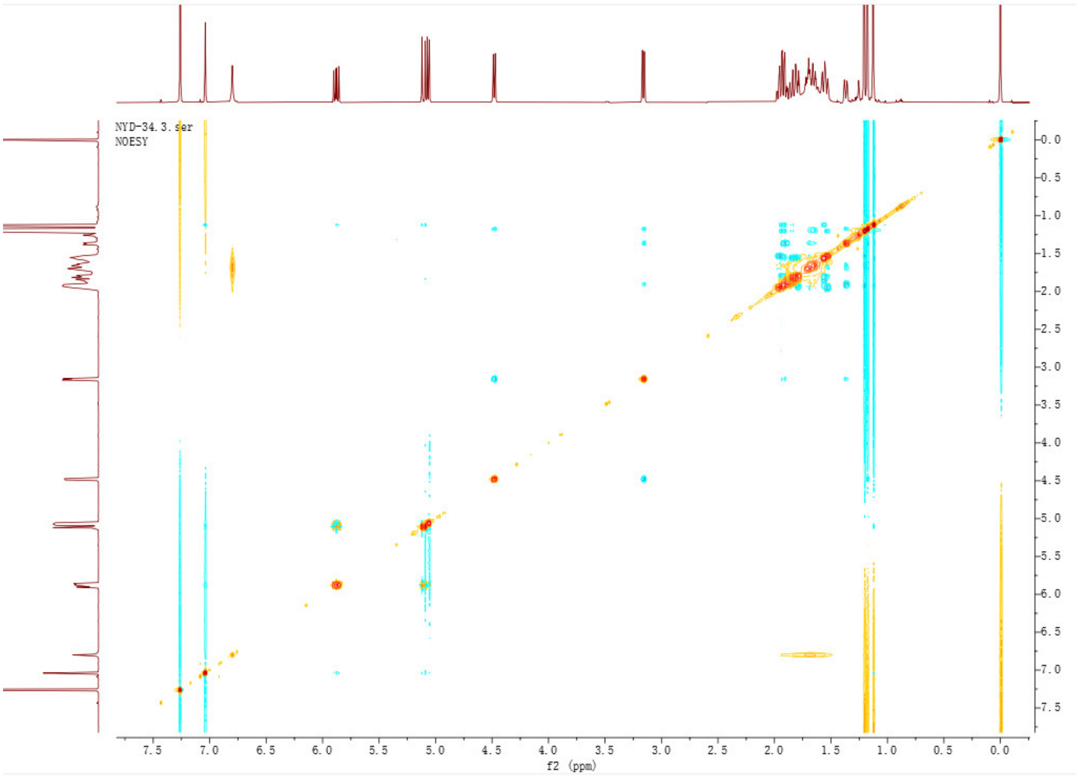

S26. HRESIMS of libertellenone Z (**3**).

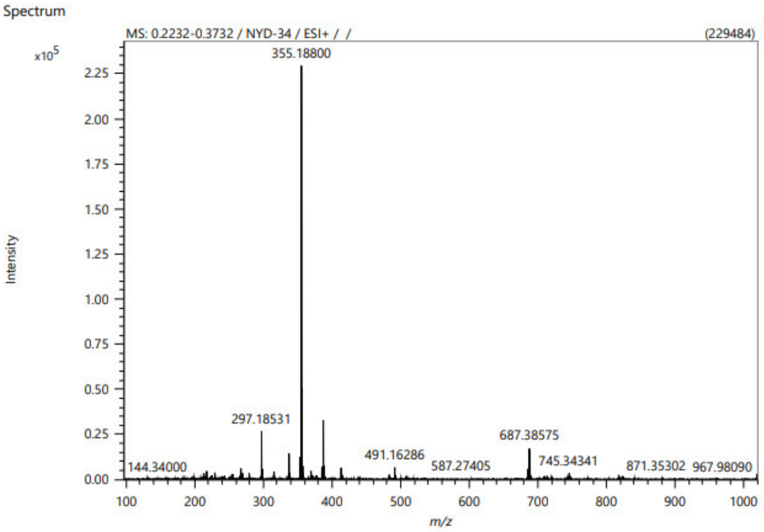

Elemental Composition

Parameters		Elements Set 1:								
Tolerance:	±5.00 ppm	Symbol	C	H	N	O	Na	S	Cl	Br
Electron:	Odd/Even	Min	0	0	0	0	1	0	0	0
Charge:	+1	Max	200	200	3	8	1	0	0	0
DBE:	-1.5 - 200.0									

Symbol	Si	F
Min	0	0
Max	0	0

Results

Mass	Intensity	Intensity [%]	Formula	Calculated Mass	Mass Difference [mDa]	Mass Difference [ppm]	DBE
355.18800	229483.69	100.00	C20 H28 O4 Na	355.18798	0.02	0.06	6.5
			C18 H26 N3 O3 Na	355.18664	1.36	3.84	7.0

S27. UV spectrum of libertellenone Z (**3**).

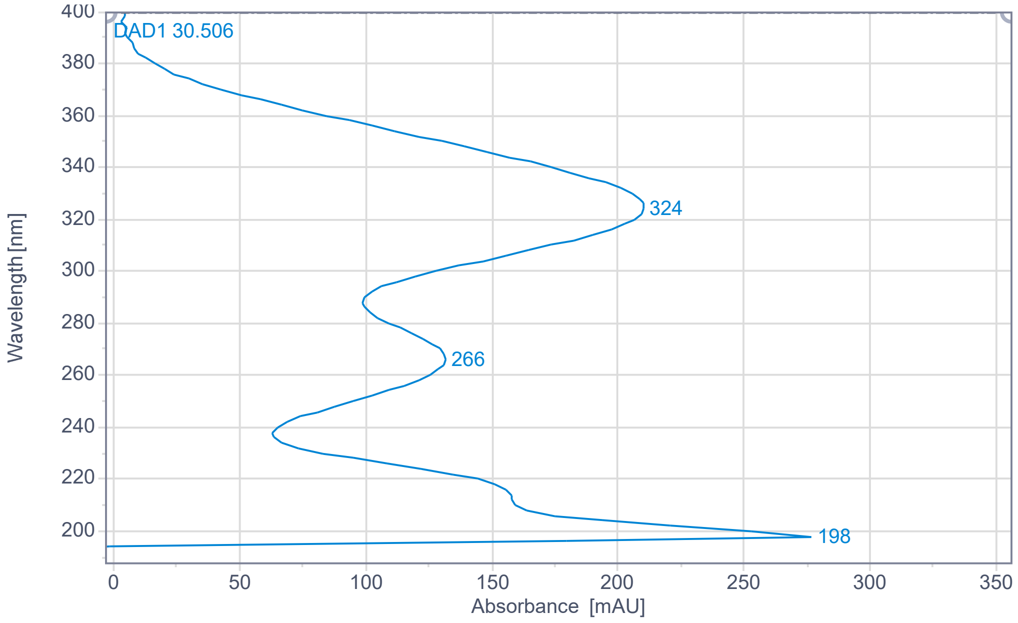

S28. ORD result of libertellenone Z (**3**)

Anton Paar MCP 5500 - Measurement Results:

Software version: 4.00.11383.92
MCP serial number: 99030100

Sample Information:

- Unique Sample Id: 50966
- Date: 2022-12-16
- Time: 14:11:51
- Method: Specific Rotation (25°C)
- Master Condition: valid
- Sample Name: NYD-34
- Concentration: 0.0500 g/100cm³
- User: student

Measurement Result:

Sub Measurement Number	Unique Sample Id	Time	Optical Rotation	Sample Cell Temperature	Specific Rotation (calc.)
			[°]	[°C]	[°]
1	50967	14:10:24	-0.0385	25.05	-76.9923
2	50968	14:10:42	-0.0390	24.97	-77.9922
3	50969	14:11:00	-0.0393	24.92	-78.5921
4	50970	14:11:32	-0.0397	24.89	-79.3921
5	50971	14:11:50	-0.0401	24.89	-80.1920
average	50966	14:11:51	-0.0393	24.94	-78.6321
std. dev.			0.000553	0.0605	1.105523

GxP Information (at 589 nm):

- Last Quartz Adjustment: 2021-11-30 15:41:39 by Administrator

S29. ^{1}H NMR spectrum of **4** in $CDCl_3$.

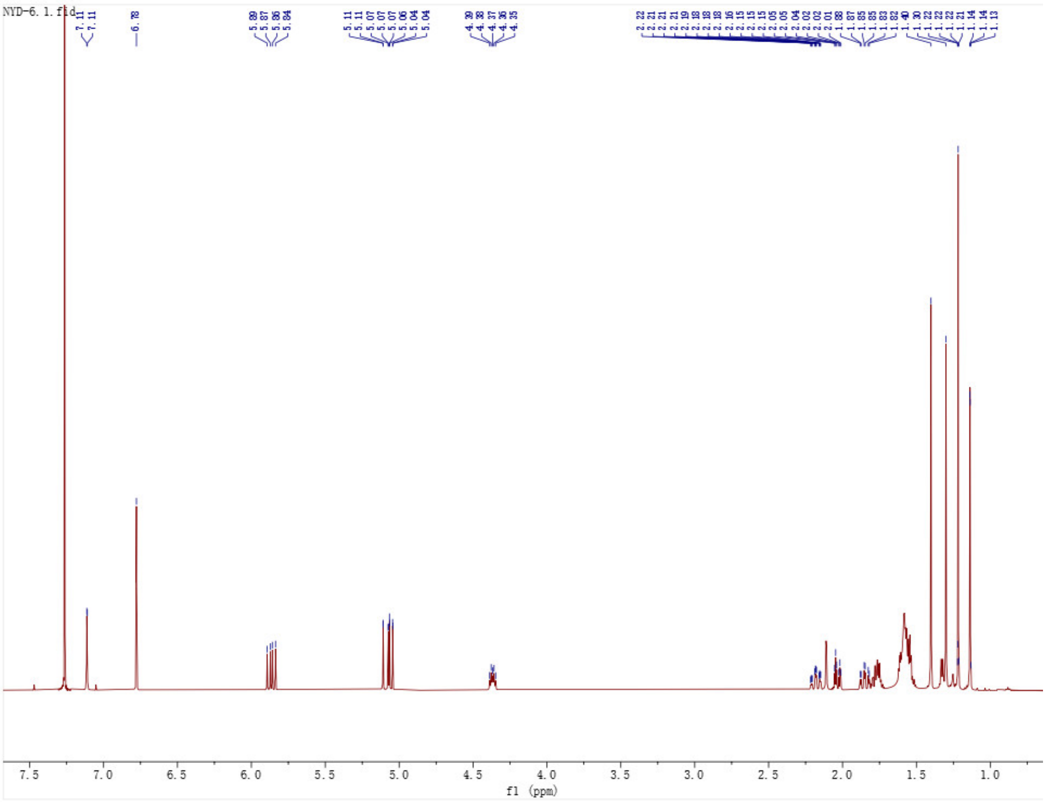

S30. ^{13}C NMR spectrum of **4** in $CDCl_3$.

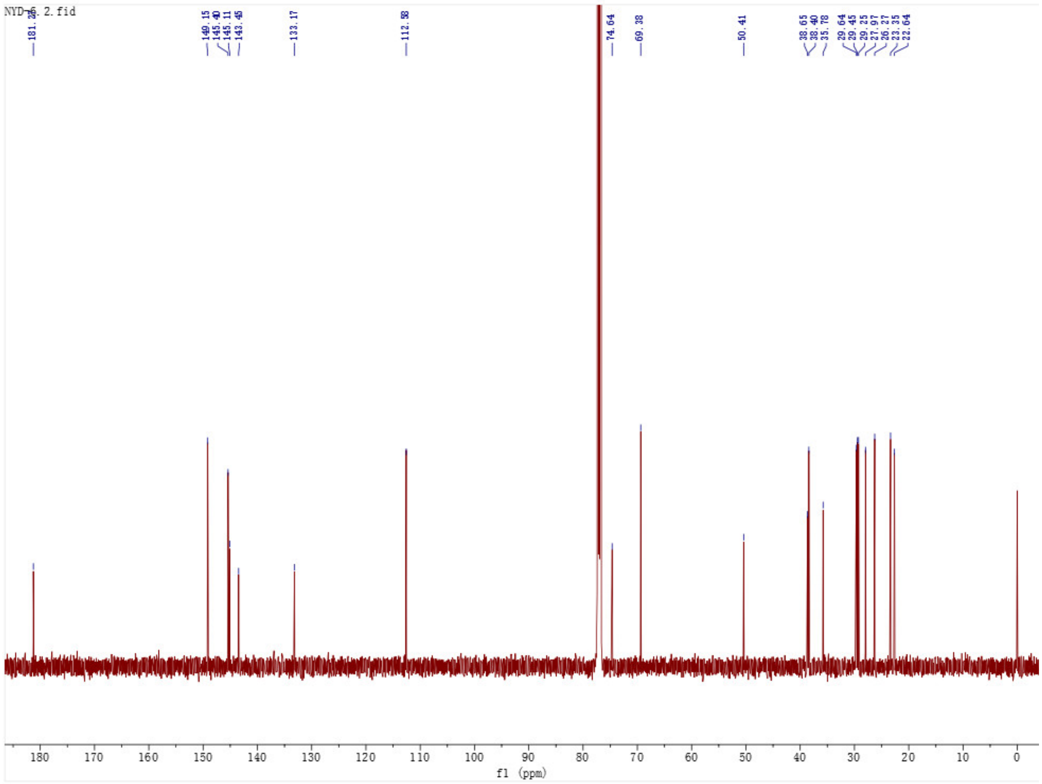

S31. ^{1}H NMR spectrum of **5** in $CDCl_3$.

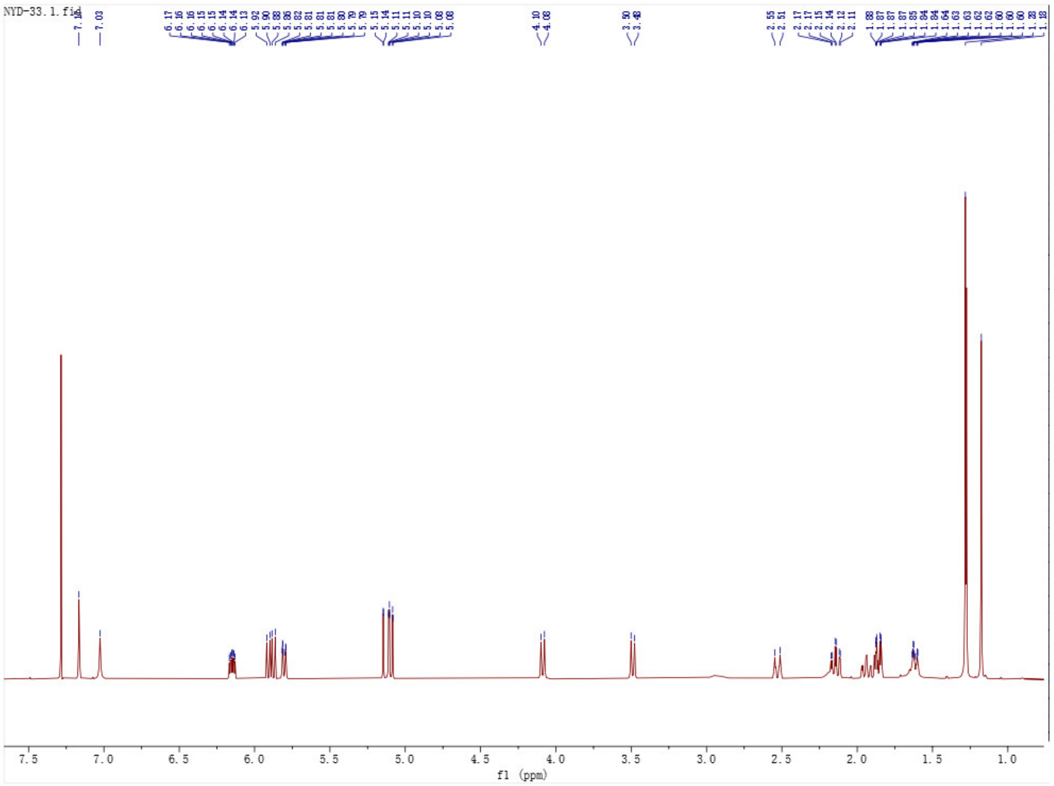

S32. ^{13}C NMR spectrum of **5** in $CDCl_3$.

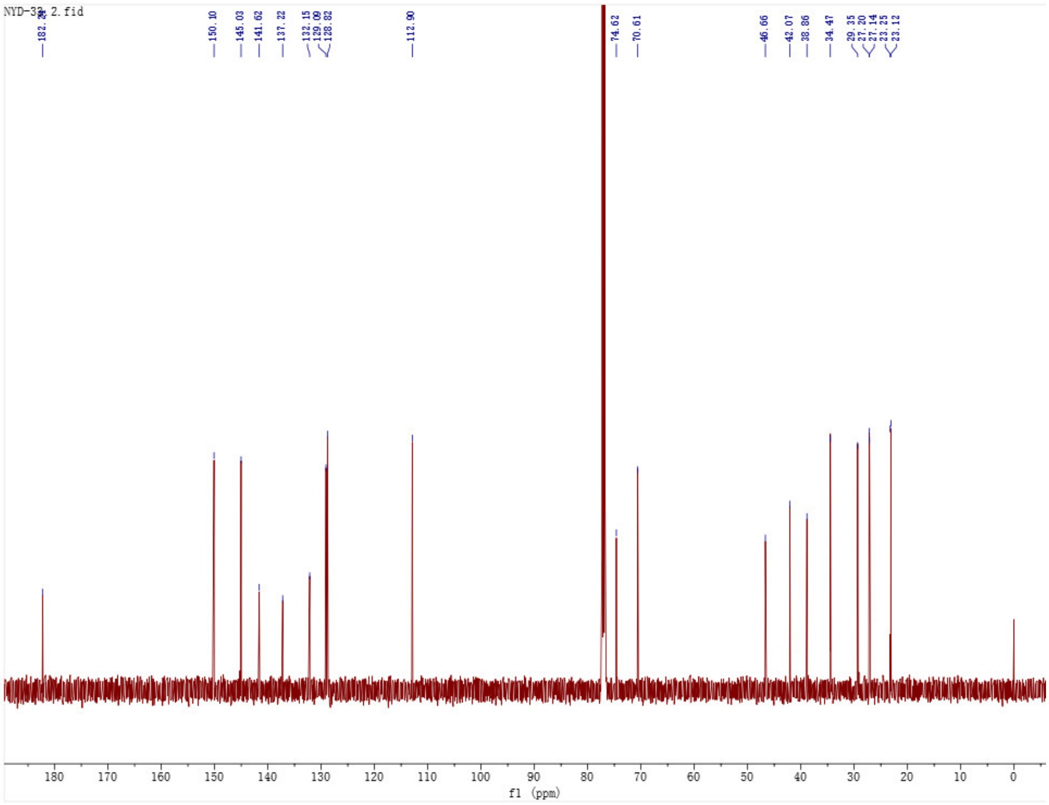

S33. ^{1}H NMR spectrum of **6** in $CDCl_3$.

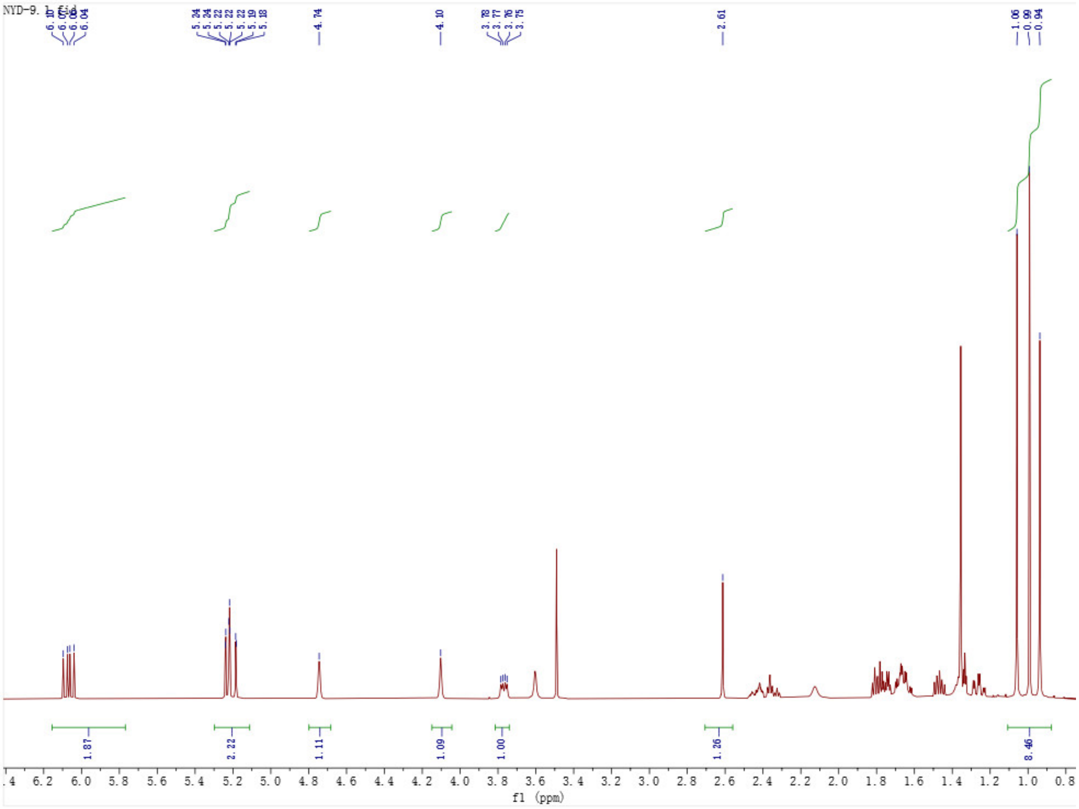

S34. ^{13}C NMR spectrum of **6** in $CDCl_3$.

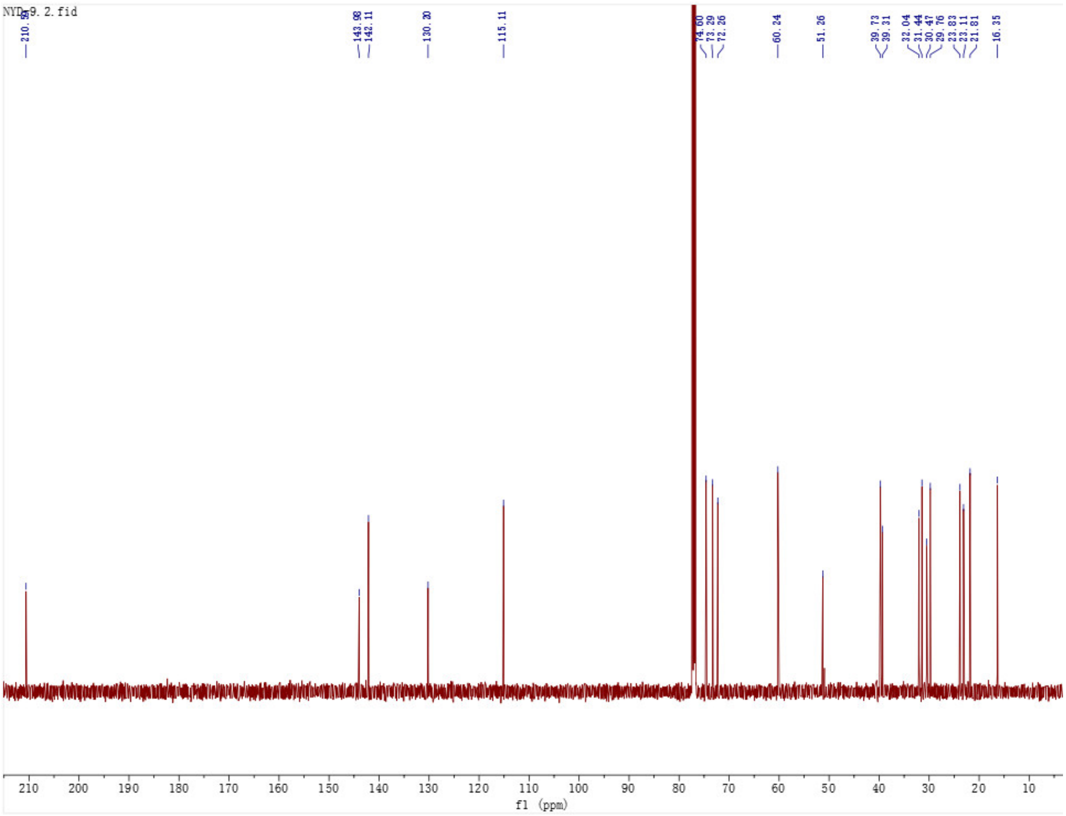

S35. ^{1}H NMR spectrum of **7** in $CDCl_3$.

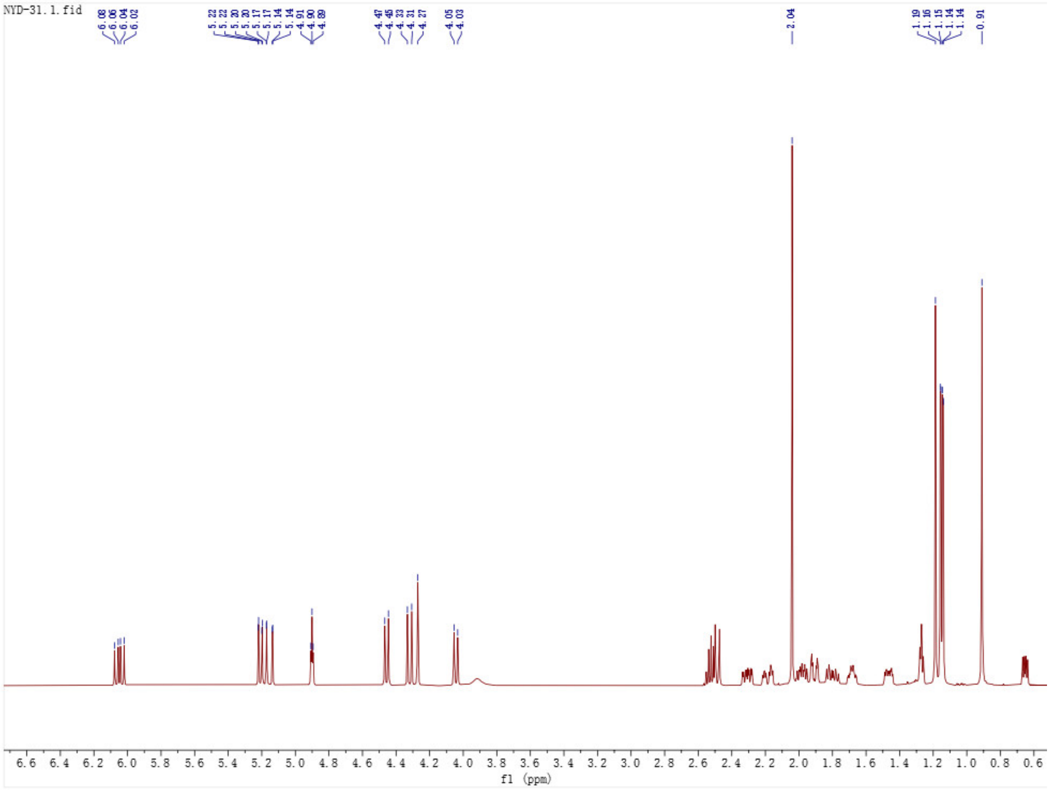

S36. ^{13}C NMR spectrum of **7** in $CDCl_3$.

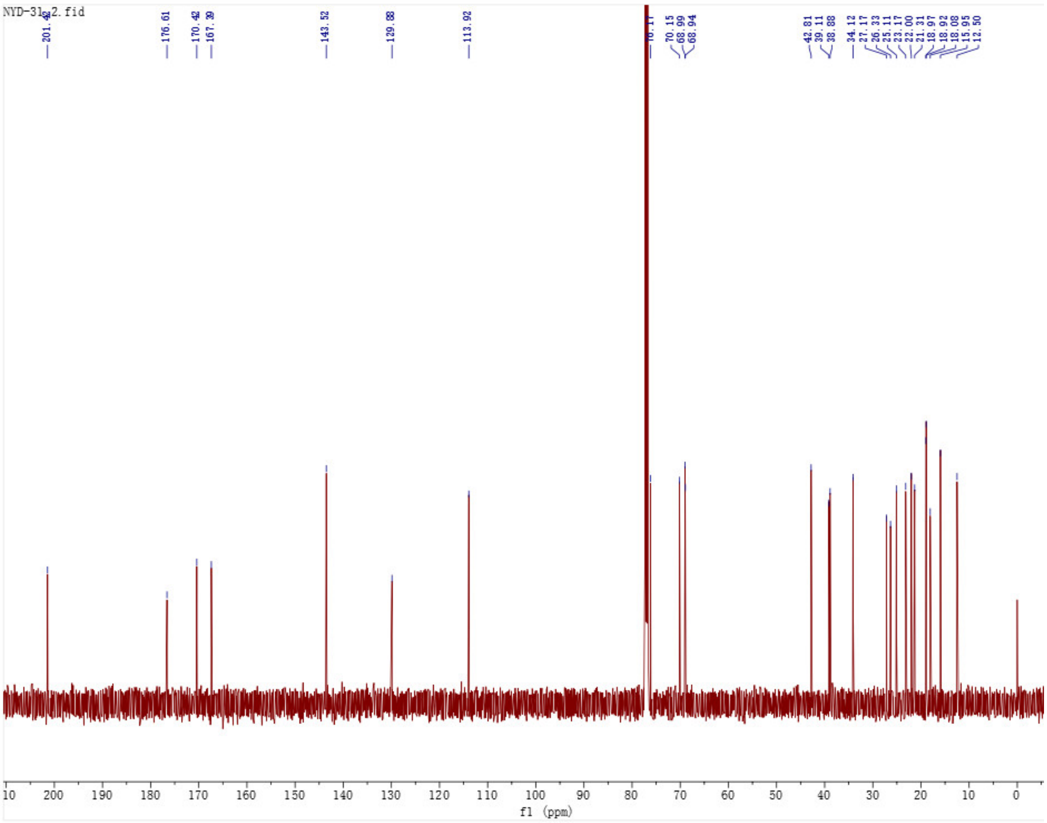

S37. The colony and mycelium characteristics of *Eutypella* sp. D-1 .

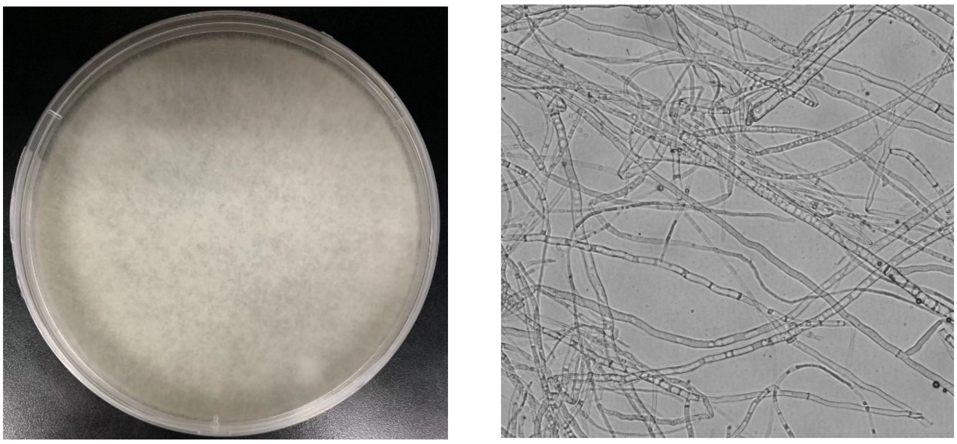

Supplement: Supplementary file 1 [file marinedrugs-21-00541-s001.zip › marinedrugs-2666747-supplementary.pdf]
